# Molecular Mechanisms Underlying Heart Failure and Their Therapeutic Potential

**DOI:** 10.3390/cells14050324

**Published:** 2025-02-20

**Authors:** Oveena Fonseka, Sanskruti Ravindra Gare, Xinyi Chen, Jiayan Zhang, Nasser Hawimel Alatawi, Claire Ross, Wei Liu

**Affiliations:** Faculty of Biology, Medicine and Health, The University of Manchester, Manchester M13 9PT, UK; oveena.fonseka@postgrad.manchester.ac.uk (O.F.); sanskrutiravindra.gare@postgrad.manchester.ac.uk (S.R.G.); xinyi.chen-2@manchester.ac.uk (X.C.); jiayan.zhang@postgrad.manchester.ac.uk (J.Z.); nasserhawimelo.alatawi@postgrad.manchester.ac.uk (N.H.A.)

**Keywords:** heart failure, hypertrophy, fibrosis, apoptosis, inflammation, metabolism

## Abstract

Heart failure (HF) is a prominent fatal cardiovascular disorder afflicting 3.4% of the adult population despite the advancement of treatment options. Therefore, a better understanding of the pathogenesis of HF is essential for exploring novel therapeutic strategies. Hypertrophy and fibrosis are significant characteristics of pathological cardiac remodeling, contributing to HF. The mechanisms involved in the development of cardiac remodeling and consequent HF are multifactorial, and in this review, the key underlying mechanisms are discussed. These have been divided into the following categories thusly: (i) mitochondrial dysfunction, including defective dynamics, energy production, and oxidative stress; (ii) cardiac lipotoxicity; (iii) maladaptive endoplasmic reticulum (ER) stress; (iv) impaired autophagy; (v) cardiac inflammatory responses; (vi) programmed cell death, including apoptosis, pyroptosis, and ferroptosis; (vii) endothelial dysfunction; and (viii) defective cardiac contractility. Preclinical data suggest that there is merit in targeting the identified pathways; however, their clinical implications and outcomes regarding treating HF need further investigation in the future. Herein, we introduce the molecular mechanisms pivotal in the onset and progression of HF, as well as compounds targeting the related mechanisms and their therapeutic potential in preventing or rescuing HF. This, therefore, offers an avenue for the design and discovery of novel therapies for the treatment of HF.

## 1. Introduction

Heart failure (HF) is a fatal progressive disorder in which defective contractile function of the myocardium leads to insufficient circulation of the blood, resulting in peripheral and pulmonary edema, hepatic and renal dysfunction, increased risk of stroke, and deterioration of cardiac function until the point of cardiac arrest [1,2,3,4]. A systematic review covering 45 epidemiological studies published between 1991–2019 determined the average prevalence of HF among adults was 3.4% [5]. There are several comorbidities and risk factors associated with HF, including cancer, obesity, hypertension, diabetes, and kidney disease [6,7,8]. HF is often classified into three major subcategories dependent upon the presence or absence of diastolic dysfunction, as determined by an echocardiogram. HF with preserved ejection fraction (HFpEF), where EF is > 50%, accounts for 50% of all HF patients and typically develops due to natural age-related cardiac decline and disorders that contribute significant degrees of systemic metabolic or inflammatory stress. A total of 35% of individuals are diagnosed with HF with reduced EF (HFrEF), where EF is below 40%, and is commonly a result of myocardial infarction (MI) or other underlying cardiovascular disorders. In recent years, a third subcategory in which EF is 40%-49%, termed HF with mid-range EF, has also been added to clinical guidelines and shares common hallmarks and etiology with both HFpEF and HFrEF [6]. HF is characterized by metabolic remodeling, cardiac hypertrophy, inflammation, fibrosis, impaired calcium (Ca^2+^) signaling, cardiac contractility, and endothelial dysfunction. However, how these pathological features present themselves varies with each HF subtype, and it is notable that cardiomyocyte death is prominent in HFrEF but not HFpEF [9,10]. There are also differences in the presentation of HF between males and females. The causes of this relate to significant hormonal variations, sexual dimorphism, and even socio-economic states. The incidence of cardiovascular death is lower in women. It could be said that females are conferred a degree of cardioprotection, largely attributed to the elevated levels of estrogen, which results in improved glucose and lipid homeostasis and reduced inflammation and fibrosis. Interestingly, as women age and have reduced estrogen levels, they become predisposed to HFpEF, further highlighting the beneficial role of this hormone [11]. Understanding the pathogenesis of HF is vital for the development of successful therapeutic strategies. In this review, we aim to discuss the principle molecular mechanisms contributing to HF and thus highlight potential targets for the treatment of this disease. 

## 2. Cardiac Structural Remodeling in Heart Failure 

Pathological cardiac structural remodeling is a pivotal hallmark of HF. In response to acute pathological stress, cardiac muscles attempt to tackle the increased workload or injury via compensated hypertrophy. Here, the ventricles thicken without hampering their volumetric capacity. A key feature of adaptive hypertrophy is that cardiac output (CO) remains unimpaired, and the observed structural changes can return to normal following the removal of the stressful stimulus [12]. However, prolonged stress induced by risk factors, including aging, diabetes, and hypertension, has deleterious effects, triggering alterations in key signal transduction and metabolic pathways and causing pathological hypertrophy, fibrosis, and apoptosis. Interestingly, many of the pathways leading to this maladaptive hypertrophy are primarily activated to promote adaptive hypertrophy, hence reinforcing the theory that the degree of myocardial pathogenic remodeling is dependent on the duration of the stress. The initial activation of these pathways can induce cardiomyocyte growth and survival, angiogenesis, antioxidant generation, and mitochondrial biogenesis. However, sustained stimulation has deleterious effects and triggers the activation of other molecular mechanisms, contributing to cell death, mitochondrial dysfunction, reactive oxygen species (ROS) production, and impaired Ca^2+^ handling [12]. In pathological hypertrophy, ventricular volume decreases in relation to the thickening of the myocardial tissue [13]. It is additionally possible for the heart to experience ventricular dilation, wherein the heart is enlarged, but the ventricular walls are thin and weak [12]. This, in part, is believed to arise due to an increase in apoptosis-driven cardiomyocyte cell death, particularly in HFrEF. Fibrosis is an important characteristic of pathological cardiac remodeling, which causes ventricular stiffness due to the accumulation of extracellular matrix proteins in the interstitial or perivascular regions of the heart, which negatively impacts the systolic and diastolic functions [14]. Additionally, in HFrEF, there is a tendency for fibrotic tissue to become deposited to replace myocardium lost due to the increased apoptosis [15]. Fibrosis not only provides mechanical stress contributing to stiffness of the myocardium but also disrupts the electrophysiology of the heart. Thus, fibrosis is a significant contributor to the development of arrhythmia [16]. Bradyarrhythmia arises via the inhibition of electrical signals derived from either the sinoatrial (SA) or atrioventricular (AV) nodes due to the increased presence of tissue with reduced conductivity [16]. Ectopy is an arrhythmogenic phenomenon in which action potentials develop and propagate from abnormal regions of the myocardium. A major risk factor for ectopy is a reduction in gap junction (GJ) coupling, which is likely due to fibrosis [16]. Finally, re-entry is the process by which electrical signals are propagated in a circular manner and trigger the continuous abnormal regeneration of actional potentials in the heart. Normally, the rate of signal propagation allows an appropriate amount of time for the myocardium to relax prior to another action potential. However, in the presence of fibrosis, electrical signals can be delayed or propagated along an abnormal pathway, causing signals to reach the relevant areas of the myocardium at the wrong time and, therefore, asynchronous and disorganized contraction [16]. This ultimately results in impaired cardiac relaxation and contractility thus significantly reducing CO [14].

## 3. Molecular Mechanisms Underlying Heart Failure 

### 3.1. Mitochondrial Dysfunction

#### 3.1.1. Mitochondrial Biogenesis in the Heart

**Figure 1:**
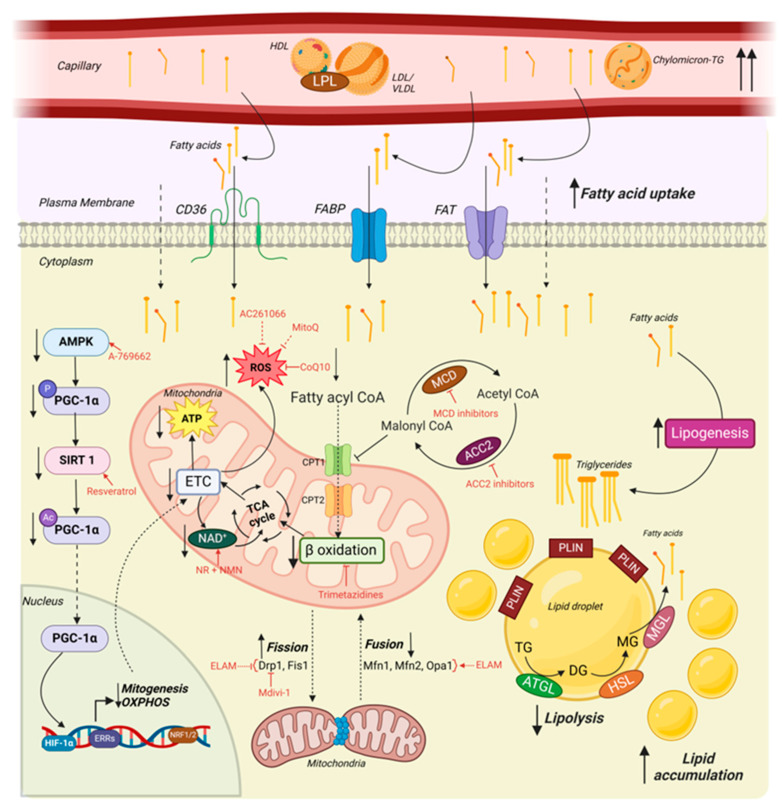
A summary of lipotoxicity and mitochondrial dysfunction in heart failure and the related treatments. Maintaining a balance between lipid uptake, usage, storage, and hydrolysis, as well as mitochondrial homeostasis and dynamics, is crucial for efficient functioning of the heart. A disturbance in this balance gives rise to harmful consequences, including lipotoxicity, leading to heart failure. Potential treatments are indicated in red. ACC2, Acetyl coenzyme A carboxylase 2; AMPK, AMP-activated protein kinase; ATGL, Adipose triglyceride lipase; ATP, Adenose triphosphate; CD36, Cluster of differentiation 36; CoQ10, Coenzyme Q10; CPT1, Carnitine palmitoyl transferase 1; CPT2, Carnitine palmitoyl transferase 1; DG, Diglyceride; DRP1, Dynamin-related protein 1; ELAM, Elamipretide; ERR, Estrogen-related receptor; ETC, Electron transport chain; FABP, Fatty acid binding protein; FAT, Fatty acid transport protein; FIS1, Mitochondrial fission protein 1; HDL, High-density lipoprotein; HIF, Hypoxia-Inducible Factor; HSL, Hormone-sensitive lipase; LDL, Low-density lipoprotein; LPL, Lipoprotein lipase; MCD, Malonyl CoA decarboxylase; MFN1, Mitofusin 1; MFN2, Mitofusin 1; MG, Monoglyceride; MGL, Monoglyceride lipase; NAD, Nicotinamide adenine dinucleotide; NMN, Nicotinamide mononucleotide; NR, Nicotinamide riboside; NRF, Nuclear Respiratory Factors; OPA1, Optic atrophy 1; OXPHOS, Oxidative phosphorylation; PGC-1α, Peroxisome Proliferator-Activated Receptor-γ Coactivator 1α; PLIN, Perilipin; ROS, Reactive oxygen species; SIRT1, Sirtuin 1; TCA, Transport electron chain; TG, Triglyceride; VLDL, Very low-density lipoprotein.

Mitochondrial biogenesis, known as mitobiogenesis, is the process by which cells increase their mitochondrial mass in response to elevated energy demands or to replace damaged mitochondria. Biogenesis requires the coordinated expression of nuclear and mitochondrial genomes, as most mitochondrial proteins are encoded by nuclear DNA, synthesized in the cytosol, and then imported into mitochondria (Figure 1) [17,18]. 

The master regulator of mitobiogenesis, peroxisome proliferator-activated receptor gamma (PPARγ) coactivator 1-alpha (PGC-1α), plays a vital role in maintaining mitochondrial homeostasis. PGC-1α typically resides in the cytoplasm in its inactive state or is bound to inhibitory factors until it is activated due to various cellular signals, including oxidative stress, fasting, and exercise. Once activated, it translocates to the nucleus, where it interacts with other co-activators and transcription factors, such as nuclear respiratory factors 1 and 2 (NRF1 and NRF2), PPAR, and estrogen-related receptor alpha (ERRα), to upregulate genes essential for mitochondrial biogenesis [19,20,21]. This includes mitochondrial transcription factor A (TFAM) for mtDNA replication, mitochondrial ribosomal proteins (e.g., MRPL12, MRPS9) for mitochondrial protein synthesis, and key components for the electron transport chain (ETC) (e.g., NDUFA, SDHB, and COX1) [22].

The activation of PGC-1α is regulated by various post-translational modifications, notably, phosphorylation and acetylation. PGC-1α phosphorylation is primarily governed by adenosine monophosphate-activated protein kinase (AMPK) [23]. As an integral component of the cellular response to energy depletion, AMPK is allosterically activated when there is elevated adenosine monophosphate/adenosine triphosphate (AMP/ATP) or creatine/phosphocreatine ratios. Such conditions arise due to metabolic stresses, including fasting, glucose deprivation, cellular stress, or oxidative stress [24,25]. Once activated, AMPK directly phosphorylates PGC-1α on threonine-177 and serine-538 positions, causing enhanced induction of the PGC-1α promoter itself and increasing its transcription [26]. It also boosts the activity and binding affinity of PGC-1α to NRF1 and PPARs, thereby promoting the expression of genes involved in mitochondrial biogenesis and fatty acid oxidation (FAO) [27]. Likewise, phosphorylation of PGC-1α by p38 mitogen-activated protein kinase (MAPK) has also been shown to regulate its activity. p38 MAPK increases the stability of PGC-1α to overcome its short half-life [28]. It also interferes with the interaction between PGC-1α and its corepressor p160 myb binding protein, which usually inhibits PGC-1α activity [29]. Consequently, PGC-1α remains active for longer periods, ensuring more effective interactions with its coactivators. Additionally, PGC-1α activity may be regulated by acetylation. While the acetyltransferase general control of nucleotide synthesis 5 (GCN5) acetylates PGC-1α, deacetylation is driven by sirtuin1 (SIRT1). As with AMPK, SIRT1 senses metabolic disturbances, stimulating its activity, which leads to increased transactivation ability of PGC-1α [30,31]. In conditions of nutrient and energy deprivation, SIRT1 detects oxidized nicotinamide adenine dinucleotide (NAD+) levels and deacetylates PGC-1α, which leads to the induction of genes regulating FAO and overall mitochondrial biogenesis [32]. Nonetheless, the precise mechanisms of how acetylation increases PGC-1α activity are not yet clear. Conversely, acetylation of PGC-1α by GCN5 inhibits its function. GCN5 directly binds to PGC-1α, triggering its relocalization from promoter regions to nuclear foci. Consequently, the expression of target genes is decreased [33,34]. 

As mentioned earlier, PGC-1α co-activates NRF1/2, which is a transcription factor regulating mitochondrial function. The previous literature demonstrates that NRF1 induces the expression of subunits assembling the five respiratory complexes of the ETC [22]. It also binds to GC-rich DNA elements within the promoter regions of genes governing mitochondrial biogenesis and respiration [35]. Moreover, NRF1 is important in sustaining mitochondrial DNA and respiratory chain function, as NRF1-null mice displayed lethality between 3.5 and 6.5 embryonic days [36]. NRF1 also induces the expression of genes, including TFAM and translocase of the outer membrane 20, which are integral players of proper mitochondrial function [37]. Likewise, NRF2 binds to the antioxidant response element in numerous mitochondria-related genes [38]. Increased nuclear localization of NRF1 and 2 has demonstrated therapeutic effects in ischemia/reperfusion (I/R)-induced HF mice by increasing antioxidant levels and preserving mitochondrial biogenesis [39]. Additionally, in a murine model of exhaustive exercise-induced cardiac injury, increased PGC-1α and NRF1/2 levels were associated with improved mitochondrial respiratory function [40]. 

In addition, the mammalian/mechanistic target of rapamycin 1 (mTOR1) is also a crucial player in preserving mitochondrial function. mTORC1, a complex containing mTOR1, is significantly involved in governing mitochondrial biogenesis, and it functions in response to energy and nutrient levels, growth factors, and stress [41,42]. Once activated, mTORC1 triggers various downstream signaling cascades modulating protein synthesis and cell metabolism. The primary downstream targets of mTORC1 are S6K1 and 4E-BP1 [43]. Numerous studies shed light on the way in which mTORC1 influences mitochondrial function. For instance, inhibition of mTORC1 by rapamycin induced a significant decrease in mitochondrial membrane potential, oxygen consumption, and oxidative capacity [44]. Additionally, the ability of mTORC1 to regulate the expression of mitochondrial genes encoded by the nuclear DNA, including ATP synthase subunit delta and TFAM, as well as the components of the ETC, has been demonstrated [45]. 

The hypoxia-inducible factor (HIF) is another key player in maintaining mitochondrial biogenesis. Hypoxia, coupled with insufficient energy production and mitochondrial dysfunction, can lead to cardiac diseases such as ischemic injury [46]. Under normoxic conditions, the HIFα subunit is hydroxylated by prolyl hydroxylases (PHDs), stimulating its degradation by the proteasome. However, under hypoxic conditions, PHDs are less active, causing HIFα accumulation. HIFα then translocates to the nucleus, where it binds to HIFβ to form the HIF complex. Consequently, HIF interacts with hypoxia response elements in the DNA to induce the expression of genes that allow for adaptation to hypoxic environments [47,48]. HIF can impact mitochondrial function in numerous ways. Firstly, HIF assists in shifting to anaerobic metabolism (glycolysis) from aerobic respiration, allowing for continuous ATP production [49]. HIFα induces the expression of relevant genes, including Glut1, to meet the increased demand for glycolysis. Further evidence from Iyer et al. demonstrates that HIFα is crucial for the expression of genes required for the metabolism of extracellular glucose into intracellular lactate. HIFα is also important for accurate cardiovascular development as HIFα knockout mouse embryos displayed developmental defects, including myocardial hyperplasia, ventricular obstruction, and pericardial effusion [50]. Moreover, O’Hagan et al. demonstrated that overexpression of PGC-1α enhances the expression of HIF-target genes through stabilizing HIFα [51]. Increasing HIFα expression reduces oxygen consumption in mitochondria dependent on complexes I, II, and IV [52]. HIFα promotes the expression of cytochrome c oxidase 4, which is an inhibitor of mitochondrial complexes [53]. This prevents unnecessary exhaustion of mitochondria in hypoxic conditions that can result in mitochondrial damage and dysfunction. 

The ERR family, composed of ERRα, ERRβ, and ERRγ, also contributes to sustaining mitochondrial homeostasis, energy metabolism, and overall cellular homeostasis. Once activated, ERRs bind to ERR response elements in the DNA and recruit coactivators or corepressors to modulate the transcription of target genes [54]. PGC-1α is one of the vital coactivators interacting with ERRs. They play a role in regulating FAO, as their increased expression has been observed in cardiac tissue, which possesses high metabolic and energy demands. ERR knockout mice displayed lower medium-chain acyl-coenzyme A dehydrogenase (MCAD) expression levels, a key enzyme in mitochondrial FAO, highlighting the involvement of ERRs in FA utilization [55]. Similarly, co-overexpression of ERRα and PGC-1α induced enhanced expression of MCAD in ventricular cardiomyocytes [56]. Notably, in a murine transverse aortic constriction (TAC) model, the level of ERRα transcripts was reduced by 33%. Similarly, ERRα null mice displayed attenuated expression of genes involved in lipid metabolism (Cd36, Asc1, and Acox1), TCA cycle (Idh2), and ETC (Cox8b, Atp5e, and Ndufb3) [57]. 

Physiologically, mitochondrial biogenesis is a finely tuned process that responds to increased energy demands, such as those induced by exercise or moderate energy stress [58,59]. This adaptive response involves the activation of PGC-1α, which enhances ATP production, mitochondrial mass, and overall cellular energy capacity—factors crucial for maintaining cardiac contractility. The increase in mitochondrial content and efficiency enables cells to meet transient energy demands effectively, preserving cellular and tissue health.

#### 3.1.2. Mitochondrial Biogenesis Impairments in Heart Failure

Pathologically, however, mitochondrial biogenesis is stimulated by adverse factors such as oxidative stress, chronic inflammation, and metabolic disturbances associated with conditions like HF and metabolic syndrome. While these stressors initially trigger biogenesis as a compensatory mechanism, they ultimately lead to an impaired biogenesis process characterized by inefficient mitochondrial function and disrupted energy metabolism. 

In humans with HF, significantly lower levels of AMPK have been observed [60]. Furthermore, PGC-1α is significantly down-regulated in the left ventricle (LV) of individuals with HFrEF or HFpEF, potentially resulting in defective mtDNA replication [61,62,63,64,65,66]. Similar impairments in mitochondrial biogenesis have been observed in dogs with HF, as discussed by Gupta et al., with a notable reduction in phosphorylation levels, indicating a dysregulation that suggests abnormal mitochondrial biogenesis [67]. This disturbance reduces mitochondrial mass and ATP synthesis, driving the energy shortage that underpins cardiac dysfunction. ERRα, a nuclear receptor that functions as a key transcriptional regulator of mitochondrial genes and acts in concert with PGC-1α to promote mitochondrial biogenesis, was also down-regulated in HF. Alterations in PGC-1α and ERRα target genes, such as Nrf1, Tfam, and Cox4, correlated with LVEF and distinguished failing from nonfailing phenotypes [68]. In addition, mice deficient in ERRγ display abnormalities in cardiac metabolic gene expression and mitochondrial function. Importantly, these mice fail to switch to FAO from glycolysis during the perinatal period, leading to newborn mortality [69]. Similarly, cardiomyocyte-specific HIF1α deletion in mice resulted in contractile deficiencies as well as alterations in the expression of genes regulating glucose metabolism and Ca^2+^ handling [70]. Also, ERRα-null mice subjected to TAC-induced pressure overload displayed HF characteristics, including reduced LV fractional shortening and chamber dilation. A reduction in ATP synthesis rates and genes modulating FAO was also noted [57]. Furthermore, it has been demonstrated that cardiac-specific NRF1 knockout mice suffered from significantly decreased LV fractional shortening and mitochondrial mass and enhanced apoptosis levels [71]. Similarly, PGC-1α and NRF2 protein levels were also diminished, indicating impaired mitochondrial biogenesis. Moreover, dietary intake of phytochemicals that increase NRF2 levels attenuated hypertension-related HF in rats. A reduction in pathological cardiac hypertrophy and fibrosis and preserved diastolic function was observed [72]. Previous studies highlight the vitality of preserving mitochondrial homeostasis as a disruption in its balance can result in detrimental consequences.

#### 3.1.3. Mitochondrial Fusion and Fission in the Heart

Mitochondria constantly undergo remodeling through mitochondrial fusion, fission, transport, and mitophagy to maintain their structural and functional integrity in signal transduction and metabolism. These processes ensure the mitochondrial network adapts to the energetic demands of cells and that the damaged and dysfunctional mitochondria are removed. 

In mammals, three key proteins mediate mitochondrial fusion: mitofusin 1 (Mfn1), mitofusin 2 (Mfn2), and optic atrophy 1 (Opa1). Mfn1 and Mfn2 regulate outer mitochondrial membrane (OMM) fusion, while Opa1 facilitates inner membrane fusion, working together to maintain mitochondrial network integrity and function (Figure 1). In mice, a deficiency in Mfn1 resulted in severely fragmented mitochondrial morphology. In contrast, Mfn2 deficiency led to a unique phenotype, where approximately 85% of mitochondria adopt spherical or oval shapes instead of their typical elongated or tubular morphology [73]. Mitochondrial fission is regulated by several key proteins, notably, dynamin-related protein 1 (Drp1) and fission protein 1 (Fis1). In mammalian cells, Drp1 and Fis1 coordinate to facilitate mitochondrial fission, with activated Drp1 forming constriction complexes on the mitochondrial membrane, while Fis1 [74,75] provides a scaffold to support complex assembly and recruitment.

#### 3.1.4. Mitochondrial Dynamic Abnormalities in Heart Failure

Dysregulation of mitochondrial dynamics is a key hallmark in HF. Abnormal mitochondrial dynamics, encompassing both fission and fusion processes, are increasingly recognized as critical contributors to cardiovascular disease (CVDs) [76]. Specifically, Drp1 has been found to be elevated in human non-ischemic dilated HF [77]. Under hyperglycemic or hyperlipidemic conditions, Drp1 was phosphorylated by Rho-associated protein kinase 1 and extracellular signal-regulated kinase 1/2 (ERK1/2), promoting its translocation to the (OMM) and initiating mitochondrial fission [78,79]. Elevated intracellular Ca^2+^ levels and reduced mitochondrial membrane potential can further drive this process. Notably, inhibiting mitochondrial fission has been shown to have protective effects against I/R injury, highlighting the importance of maintaining the balance between fission and fusion in cardiac health [76].

Disruptions in mitochondrial fission and fusion dynamics in HF are closely tied to Ca²⁺ dysregulation. Elevated cytosolic Ca²⁺ levels, a hallmark of HF, stimulate the phosphatase calcineurin [80], which dephosphorylates Drp1 at the Ser 637 residue [81]. This modification activates Drp1 and facilitates its translocation to the OMM where it promotes mitochondrial fission. Excessive fission resulting from persistent Drp1 activation contributes to mitochondrial fragmentation, impaired ATP production, and increased oxidative stress—factors that exacerbate cardiomyocyte dysfunction and accelerate HF progression [82]. This process illustrates how Ca²⁺ homeostasis and mitochondrial dynamics are linked in the pathophysiology of HF. 

#### 3.1.5. Therapeutically Targeting Mitochondrial Biogenesis and Dynamics in Heart Failure

Given the critical role of AMPK in regulating genes involved in mitochondrial biogenesis, restoring or fully activating AMPK activity in this context may offer therapeutic benefits. A-769662, a compound activator of AMPK, has been shown to preserve mitochondria in the heart, thereby attenuating myocardial I/R injury in mice [83,84]. As previously mentioned, SIRT1 is another activator of PGC-1α. Resveratrol, a phytoalexin naturally produced by certain plants, activates SIRT1 and has been shown to mitigate cardiac injuries in diabetic cardiomyopathy (DCM) through PGC-1α-mediated mitochondrial regulation [85].

Mdivi-1, a mitochondrial fission inhibitor-1, is the first selective inhibitor of the mitochondrial fission protein Drp1 and has shown cytoprotective effects in various cardiovascular injury models [86]. It effectively reduces mitochondrial fragmentation and apoptosis, helping to restore the crucial balance between fission and fusion. Similarly, elamipretide (ELAM) shows promise in treating HF by restoring the balance between mitochondrial fusion and fission, enhancing fusion through upregulation of MFN1, MFN2, and OPA1 and inhibiting fission by downregulating Fis1 and DRP1 [87]. A notable study found that a 3-month monotherapy with ELAM improved LV systolic function and prevented progressive LV dilation [88]. Together, these agents represent innovative approaches to targeting mitochondrial dynamics and improving cardiac health.

#### 3.1.6. Cardiac Energy Production in the Heart

The heart is a metabolically demanding organ, relying heavily on a constant supply of ATP to fuel its contractile function. Mitochondria are the primary source of ATP, which produce more than 95% of the ATP in the myocardium and account for nearly 30% of cardiomyocyte volume. Under normal physiological conditions, mitochondria generate ATP through oxidative phosphorylation (OXPHOS), a process whereby substrate molecule acetyl-CoA is metabolized to produce reduced nicotinamide adenine dinucleotide (NADH) and oxidized flavin adenine dinucleotide (FADH₂) through the tricarboxylic acid (TCA) cycle, also known as the Krebs cycle (Figure 1). 

The main substrates used by cardiomyocytes are fatty acids (FAs) and glucose, which undergo β-oxidation and glycolysis, respectively [89], to provide acetyl-CoA for the TCA cycle. The resultant high-energy electrons carried by NADH and FADH_2_ are subsequently transferred to the ETC, driving ATP synthesis. This process is tightly coupled to mitochondrial oxygen consumption, as oxygen serves as the terminal electron acceptor at Complex IV in the ETC. Oxygen consumption reflects the rate of electron transfer through the ETC and is essential for maintaining the proton gradient that drives ATP synthesis via ATP synthase. However, oxygen consumption alone, whether measured in a whole-organism or tissue-specific level, is not a direct measure of ATP production. The efficiency of ATP generation per unit of oxygen consumed can vary significantly due to proton leak, variations in substrate utilization, or uncoupling of the ETC from ATP synthase [90,91].

#### 3.1.7. Metabolic Shifts in Heart Failure

In adults, the heart primarily depends on FAO for energy production, with glucose metabolism serving a supportive role. However, in HF, particularly under metabolic stresses like diabetes or obesity, there is often a shift away from efficient FAO toward glycolysis. Furthermore, an intriguing risk factor for the development of CVDs is the presence of cancer. Much research has been conducted, and it has been revealed that crosstalk between cancerous tissue and the heart can exacerbate both diseases [8]. For example, the cancer-associated increase in ataxin-10 reduced cardiac FA metabolism [92]. In HFrEF, FAO declines, and cardiac metabolism shifts to rely more on glucose, with a notable increase in glycolysis [93]. However, in a normal adult heart, ATP from glycolysis contributes less than 5% of the total ATP consumed. Thus, upregulating glycolysis alone does not effectively enhance energy supply, ultimately contributing to mitochondrial dysfunction and energy depletion [94,95]. This shift is partly due to the downregulation of key enzymes in the FAO pathway [96], limiting the heart’s ability to effectively oxidize FAs. Conversely, some recent studies have proposed that in HFpEF, cardiac FAO may be increased, shifting the balance of substrate utilization in the heart [97]. Increased expression of malonyl-CoA decarboxylase (MCD) in HFpEF reduces malonyl-CoA levels, thereby promoting FA uptake and β-oxidation [96]. Additionally, in DCM, levels of PPARα, a key transcriptional regulator of genes involved in FA metabolism, are elevated. This enhanced reliance on FAO in HFpEF may occur alongside a reduction in insulin-stimulated glucose oxidation [96], reducing overall ATP yield and leading to an energy deficit, further complicating the metabolic landscape in this condition. Controversially, recent studies on impaired myocardial energetics in HFpEF hearts indicated that the issue was not primarily due to a switch to alternative substrates but rather due to inadequate FAO [97]. This conclusion was supported by clinical evidence demonstrating a significant reduction in FAO protein levels in HFpEF patient hearts [98]. Together, these findings indicated that targeting cardiac metabolic pathways could be a promising therapeutic strategy for treating HF.

#### 3.1.8. Oxidative Stress in Heart Failure

Oxidative stress also contributes to myocardial tissue damage [99], which is evident in both the myocardium and plasma and is closely associated with LV dysfunction in patients with HF [100]. Oxidative stress is a condition that arises when there is a disruption in the balance between the production of ROS or free radicals and the body’s antioxidant defenses, leading to ROS accumulation and subsequent cellular damage [101]. Mitochondria are the main source of ROS in cardiomyocytes, through complex I and II, as most ATPs are generated through OXPHOS in the ETC. Under normal physiological conditions, mitochondria are protected from oxidative damage by a range of antioxidant defense systems [102], including enzymes such as superoxide dismutase (SOD), catalase (CAT), and glutathione (GSH) peroxidase (GPX). These antioxidants neutralize ROS, converting them into less reactive molecules like water, thus preventing the accumulation of oxidative damage, which disrupts mitochondrial function. Excessive ROS formation or inadequate mitochondrial antioxidant defenses lead to oxidative damage of essential biomolecules and cause the release of pro-apoptotic proteins, such as cytochrome *c*, from the mitochondrial intermembrane space into the cytosol [103], leading to apoptosis and further myocardial injury. 

Despite the clear role of oxidative stress in HF, clinical trials using conventional antioxidants, such as vitamin E and N-acetylcysteine (NAC), have largely failed to provide therapeutic benefits. The Heart Outcomes Prevention Evaluation study found that vitamin E supplementation did not significantly improve cardiovascular outcomes over a 4.5-year follow-up period [104]. Likewise, although NAC has shown potential benefits for HF in preclinical models, it failed to demonstrate therapeutic efficacy in patients with doxorubicin-induced cardiomyopathy [105,106]. The failure of these antioxidants in clinical settings may be attributed to their inability to selectively target mitochondria, where oxidative stress is most pronounced in HF. ROS also plays critical roles in physiological redox signaling, including mitochondrial quality control and adaptive stress responses. Therefore, broadly scavenging ROS may disrupt essential cellular processes rather than providing cardioprotection. Moreover, most antioxidant defense mechanisms in cells rely on enzymatic systems rather than small-molecule scavengers. Given these limitations, therapeutic strategies that enhance endogenous antioxidant enzyme activity or directly target mitochondrial oxidative stress are emerging as more promising alternatives in HF treatment. 

#### 3.1.9. Targeting Metabolic and Oxidative Stress in Heart Failure 

In recent years, numerous studies have explored the role of SIRT3 in HF and its potential as a therapeutic target. SIRT3, as a NAD⁺-dependent deacetylase, becomes activated in response to high NAD⁺ levels, allowing it to deacetylate and modulate key mitochondrial enzymes, which enhances FAO, the TCA cycle, and the antioxidant defense. SIRT3 deacetylates multiple proteins involved in oxidative stress resistance, enhancing their activity and promoting mitochondrial antioxidant defense. Notably, a study demonstrated that manganese SOD2 (MnSOD2), a key mitochondrial antioxidant enzyme, was directly activated by SIRT3 through deacetylation [107], enhancing its ability to neutralize superoxide radicals. In addition to its direct action on MnSOD2, SIRT3 also facilitates the nuclear translocation of Forkhead box protein O3 (FoxO3a) via deacetylation in the cytoplasm. This transcription factor regulates the expression of MnSOD2 and CAT, further strengthening the antioxidant defense system against mitochondrial oxidative stress [108,109,110,111]. As an antioxidant, mitochondrial GSH also plays a crucial role in mitigating the damaging effects of mitochondria-generated ROS by detoxifying hydrogen peroxide via the GPx system, where reduced GSH is converted into its oxidized form (GSSH). GSH reductase regenerates GSH from GSSG using NADPH as a cofactor. SIRT3 enhances this process by deacetylating glutamate dehydrogenase, which promotes NADPH production, thereby increasing the availability of GSH for GPx activity [112]. Although no drugs currently target SIRT3 directly in clinical settings, research is exploring compounds that upregulate SIRT3, given its therapeutic potential in HF and other diseases. NAD⁺ is a crucial cofactor for members of the SIRT family, which plays important roles in regulating cellular metabolism, inflammation, and mitochondrial function. Recent interest in NAD⁺ biology has spotlighted NAD⁺ precursors like nicotinamide riboside (NR) and nicotinamide mononucleotide (NMN), which boost cellular NAD⁺ levels across various tissues, often yielding therapeutic effects. These NAD⁺ precursors support SIRT3 activation indirectly. Studies have shown that increasing NAD⁺ levels through NR supplementation can blunt the worsening of cardiac function [113], while NMN supports recovery in conditions linked to NAD⁺ depletion, showing the potential to improve heart health [114]. Beyond mitochondrial metabolism, supplemented NAD⁺ also benefits the inflammatory response via SIRT1 and SIRT3 activation. SIRT1 deacetylates and inhibits nuclear factor kappa-light-chain enhancer of activated B cells (NF-κB) [115], a key regulator of pro-inflammatory cytokines. Meanwhile, as mentioned earlier, NAD⁺ promotes FoxO3a deacetylation, which upregulates autophagy-related genes, including Bcl-2 interacting protein 3 and ATG8/Microtubule-associated Protein 1 Light Chain 3 (LC3) [116]. Furthermore, SIRT3 has been shown to promote the expression of PTEN-induced kinase 1 (PINK1) [117], a key initiator of mitophagy, thereby facilitating the removal of dysfunctional mitochondria and protecting against cardiac stress-induced mitochondrial damage, thus enhancing mitophagy.

Coenzyme Q10 (CoQ10) is a naturally occurring antioxidant that has been reported to show benefits in lowering ROS levels, ultimately improving LV diastolic function [118]. Metanalyses of clinical trial data have indicated promising effects of CoQ10 administration as an HF treatment with individuals displaying increased EF and CO. However, they have concluded that the efficacy of this therapeutic strategy was only sufficient in those with a less severe degree of cardiac dysfunction [119,120,121]. Other bioactive compounds with significant antioxidant properties, such as flavonoids, carotenoids, and resveratrol, have also been found to contribute to the protection against oxidative stress in CVDs [122].

MitoQ has demonstrated beneficial effects across various animal models of diseases, including cardiovascular disease. Its mitochondrial-targeted antioxidant activity is attributed to its ability to scavenge superoxide, peroxyl, and peroxynitrite ROS upon accumulation in mitochondria [123,124,125]. Notably, this antioxidant capacity is recyclable—once oxidized, MitoQ can be regenerated into its active ubiquinol form through the ETC, ensuring sustained ROS neutralization and mitochondrial protection. SkQ1 is another mitochondrion-targeted antioxidant with activity similar to MitoQ. A study in a mouse model demonstrated that SkQ1 effectively improved mitochondrial function and cellular respiration [126].

Finally, the use of retinoic acid receptor (RAR) β agonists has proven to be potentially useful for the treatment of HF. RARβ is a nuclear receptor that interacts with retinoic acid and plays a significant role in cardiac development. Ligand binding of the receptor allows for the transcription of downstream target genes. One such effect is the respective increase and decrease in anti-oxidative and oxidative proteins. In a murine HF model, mice treated with the RARβ agonist AC261066 exhibited a significant decrease in MI-induced systolic dysfunction and oxidative stress. However, despite these promising data, its use in humans requires investigation [127].

## 4. Cardiac Lipotoxicity

### 4.1. Lipid Metabolism in the Heart 

Owing to the need to sustain continuous contraction of the myocardium, the heart is a highly energy-demanding organ. Oxidation of FAs provides approximately 70% of the ATP to meet these metabolic requirements [128]. FAs commonly derive from triglycerides (TGs) contained in chylomicrons or lipoproteins that are hydrolyzed by lipoprotein lipases extracellularly to provide free FAs. Alternatively, free FAs bound to albumin can be utilized for the oxidation of FAs. Firstly, free FAs are taken up into cardiomyocytes by passive diffusion or via the transporters, FA translocase cluster of differentiation 36 (CD36), FA binding protein, or FA transport proteins [129,130]. Once in the cytosol, the free FAs are esterified into fatty acyl CoA, translocated into mitochondria in a reaction catalyzed by carnitine palmitoyl transferase (CPT) 1 and 2, and finally, enter the β-oxidation cycle to produce ATP (Figure 1) [131]. 

Some cytosolic FAs are esterified to form TGs by a process termed lipogenesis. Initially, in lipogenesis, glycerol is converted to glycerol-3-phosphate by glycerol kinase. Then, an acyl CoA is added to the first carbon of glycerol-3-phosphate to form lysophosphatidate, catalyzed by glycerol-3-phosphate acyltransferase (GPAT) [132,133]. This is followed by the addition of acyl CoA onto the second carbon, catalyzed by acylglycerolphosphate acyltransferase (AGPAT) to form phosphatidate [134]. Subsequently, phosphatidic acid phosphatase catalyzes the conversion of phosphatidate into diacylglycerol (DG). Then, diacylglycerol acyltransferase (DGAT) drives the formation of TGs by the addition of an acyl CoA molecule [135]. These TGs can be stored in the form of lipid droplets (LDs) that are coated with the surface proteins called perilipins (PLINs) [129]. These LDs act as a reservoir of energy sources to be accessed according to the metabolic demands of the myocardium. TGs stored intracellularly in LDs are hydrolyzed by a process termed lipolysis. Lipolysis is sequentially carried out by adipose triglyceride lipase (ATGL), hormone-sensitive lipase, and lastly, monoglyceride lipase (Figure 1) [136,137].

### 4.2. Cardiac Lipotoxicity in Heart Failure

A fine physiological equilibrium between lipid uptake, utilization, and storage is essential in maintaining metabolic homeostasis in the heart. Disruption of this balance can result in cardiac lipotoxicity, which has been implicated in many cardiac abnormalities [138,139]. For instance, a study by Sharma et al. demonstrated that non-ischemic HF patients suffered from impaired lipid metabolism, evidenced by intramyocardial lipid overload and dysregulated expression of lipid-associated proteins. Similar results were obtained from Zucker diabetic rats with contractile dysfunction associated with cardiac lipid overload [140]. Concurrently, increased cardiac lipid content correlated with enhanced LV mass and reduced septal wall thickening, emphasizing the effect impaired lipid metabolism may exert on cardiac function [141]. Likewise, abnormal intramyocardial lipid accumulation has been shown to be closely linked to DCM, leading to cardiac dysfunction [142].

Intramyocardial lipid accumulation can arise due to an increase in TG synthesis, lower lipolysis rates, impaired FAO, or a combination of all. Animal models of lipotoxicity give us an insight into the molecular mechanisms that might be driving cardiac dysfunction by providing an understanding of potential causes of lipotoxicity. GPAT-knockout mice administered with a high sucrose or high-fat diet displayed significantly reduced myocardial TG levels in comparison to wild-type mice [143]. In accordance, cardiomyocyte-specific DGAT overexpressed transgenic mice developed cardiomyopathy and diastolic dysfunction that resulted from increased cardiac lipid accumulation and fibrosis [144]. These studies highlight that dysregulated lipogenesis is a way in which hyperlipidemia arises in cardiomyocytes. 

Furthermore, impaired lipolysis is another significant contributor to cardiac steatosis. For instance, ATGL knockout mice displayed cardiac lipid accumulation, cardiac dysfunction, and premature death. It appears that this occurred as a consequence of decreased PPARα and PPARδ target genes observed in ATGL null mice, leading to diminished PGC1α and PGC1β levels. The result is a compromised mitochondrial FAO and energy production. In the same study, we learned that treating ATGL knockout mice with a PPARα agonist fully reverses the detrimental phenotypes observed in the knockout mice [145]. In line with this, a study by Kienesberger et al. revealed that cardiomyocyte-specific ATGL knockout mice exhibited markedly higher levels of TG accumulation, cardiac fibrosis, and hypertrophy, which was accompanied by cardiac dysfunction [146]. The LD surface protein PLIN5 inhibits ATGL activation by binding to its co-activator comparative gene identification-58, consequently blunting lipolysis [147]. In support of this, cardiac-specific PLIN5 overexpressed transgenic mice displayed chronic lipid accumulation, leading to cardiac hypertrophy and dysfunction. A lack of PPAR target genes was also noted [148,149]. Conversely, PLIN5 null mice exhibited more efficient FAO levels, utilizing FAs and diminishing lipid accumulation in the heart [150]. These studies highlight the importance of preserving a good balance between the storage and usage of FAs to avoid lipotoxicity and subsequent cardiac abnormalities. 

The recent literature suggests that increasing FAO rates may aid in averting lipotoxicity by preventing excess lipid accumulation. A supporting study by Cheng et al. demonstrated that cardiac-specific deletion of PPARδ attenuated the expression of FAO-related genes and, therefore, reduced myocardial FAO rates. This induced myocardial lipid accumulation and cardiac hypertrophy, followed by congestive HF in mice [151]. Moreover, prolonged inhibition of CPT1 using Etomoxir led to a significantly higher accumulation of intramyocellular lipids caused by a suppression of FAO [152]. Further evidence for this arrives from a cardiac-specific acetyl coenzyme A carboxylase 2 (ACC2) knockout mice model. Physiologically, ACC2 inhibits FAO by preventing the entry of fatty acyl CoA into mitochondria; therefore, a deficiency of ACC2 increases FAO. Upon 24 weeks of high-fat diet feeding, ACC2 knockout mice exhibited diminished pathological cardiac remodeling and mitochondrial dysfunction, hence a better cardiac function [153]. These findings signify the vitality of sustaining an equilibrium between lipid availability and utilization, especially when nutrients are abundant, as in obese conditions. 

A few clinical studies have also provided insight into the way in which mutations in lipid metabolism-associated genes can impact lipid homeostasis. For instance, deletions, substitutions, and insertions in the CD36 gene can result in either type I or type II CD36 deficiency. Type I deficiency is characterized by a loss of CD36 in both monocytes and platelets, while in type II, CD36 is deficient only in platelets but present in monocytes [154]. Lower levels of cardiac FA accumulation have been observed in subjects affected by these deficiencies [155]. Similarly, a study by Yanai et al. also revealed significantly elevated serum cholesterols and low-density lipoprotein receptors, indicating a lack of lipid intake [156]. Adding to this, mutations in the AGPAT2 genes are associated with the development of lipid metabolism disorders, including congenital generalized lipodystrophy, which is characterized by hyperlipidemia and early development of diabetes [157]. In a similar manner, mutations in the PLIN1 genes, including c.1210-1GT and c.1191-1192delAG, are linked to hypertriglyceridemia, steatosis, and insulin resistance [158]. Human studies indicated the importance of maintaining proper lipid metabolism; however, there is a great need for studies investigating the underlying molecular mechanisms for potential clinical applications. 

An increasing number of studies have shed light on toxic lipid intermediates such as ceramides and DGs as being one of the drivers of cardiac lipotoxicity. Ceramides are a class of lipid molecules that regulate many physiological processes, including metabolism and inflammatory responses [159]. The levels of ceramides have been shown to increase with stress conditions, and elevated myocardial ceramide levels have been observed in failing human hearts [160]. A supporting study demonstrated that the inhibition of serine palmitoyl transferase, an enzyme involved in ceramide biosynthesis, preserved systolic function and increased survival rates [161]. Furthermore, a deficiency of stearoyl-CoA desaturase activity, the rate-limiting enzyme catalyzing the biosynthesis of monosaturated FAs, exhibited protective effects by correcting heart dysfunction observed in spontaneous obese mice (*ob*/*ob*). This was due to the lowering of toxic lipid species levels, including ceramides and DGs [162]. In a similar way, DGs act to stimulate the protein kinase C (PKC) group of enzymes, which phosphorylate proteins at serine and threonine residues. This leads to the phosphorylation of PKC substrates, including insulin receptor substrate 1, NF-κB, Ca^2+^ channels, and titin. The result was the induction of insulin resistance, oxidative stress, cell death, and inflammation, ultimately leading to cardiac dysfunction [163]. The above studies indicate a critical role for toxic lipid intermediates in the pathogenesis of lipotoxic cardiomyopathy. 

### 4.3. Targeting Abnormal Lipid Metabolism in Heart Failure

Over recent years, various pharmaceutical approaches have been developed in an attempt to tackle cardiac lipotoxicity. In particular, trimetazidines are among them. They act to increase the efficiency of energy supply by reducing FA oxidation rates via inhibition of 3-ketoacyl-CoA thiolytic enzyme. As a result, glucose is stated as the preferred fuel source for ATP production [164]. A clinical trial of HF patients revealed that treatment with trimetazidines improved LV function, consequently leading to better exercise tolerance and quality of life. Trimetazidines are approved to be used clinically to treat anginas in Europe [165].

Moreover, since malonyl CoA inhibits the action of CPT-1, MCD inhibitors are another novel approach developed to tackle lipotoxicity driving HF. MCD physiologically functions to convert acetyl CoA to malonyl CoA; therefore, an inhibition of MCD increases malonyl CoA levels. This reduces the uptake of FA into mitochondria [166]. MCD inhibition has been shown to improve cardiac function, contractility, and enhanced cardiac antioxidant capacity in failing rat hearts [167]. Also, in ischemic pig hearts, MCD inhibition led to blunted lactate production, coupled with an increased shift to glucose metabolism for ATP production [168]. 

Furthermore, ACC2 inhibitors are a novel class of drugs that have demonstrated therapeutic potential by their effectiveness in treating cardiac lipotoxicity. This is achieved by enhancing FAO, which leads to a lessened dependency on glucose metabolism for energy production [169]. For instance,, the administration of the highly selective ACC2 inhibitor, TLC-3595, in ATGL knockout mice significantly reduced cardiac TG content while increasing FAO and TCA metabolites. Consequently, TLC-3595-treated ATGL knockout mice displayed improved EF compared to untreated mice [170]. Such promising results from cardiac ACC2 knockout and inhibitor studies highlight the need for further preclinical and clinical studies to confirm findings and determine long-term safety and side effects (Figure 1). 

Taken together, emerging evidence demonstrates that maintaining a balance between lipid uptake, storage, synthesis, and utilization has treatment potential for HF. A better and deeper understanding of the molecular interplay of lipid metabolism is still required for more effective therapeutic options to be developed. 

## 5. Protein Quality Control

### 5.1. Endoplasmic Reticulum and the Unfolded Protein Response in the Heart

The endoplasmic reticulum (ER) is an interconnected network of membrane-like structures. It is an organelle of utmost importance due to its role in protein translation, post-translational modification, vesicular trafficking of proteins, Ca^2+^ homeostasis, and lipid biosynthesis [171]. Various physiological and pathological insults, including hypoxia, oxidative stress, Ca^2+^ depletion, energy deprivation, DNA damage, and altered glycosylation, can trigger improper protein folding, leading to ER stress [172]. In response to ER stress, cells elevate their protein folding capacity and machinery, terminate protein synthesis, and activate the ER-associated protein degradation (ERAD) pathway. These responses are products of the unfolded protein response (UPR), consisting of three major signaling branches governed by the transmembrane proteins, inositol-requiring protein 1 (IRE1), double-stranded RNA-dependent protein-kinase-like ER kinase (PERK), and activating transcription factor 6 (ATF6). Prolonged and unresolvable ER stress triggers apoptosis to dispose of unhealthy cells beyond repair [173,174].

**Figure 2:**
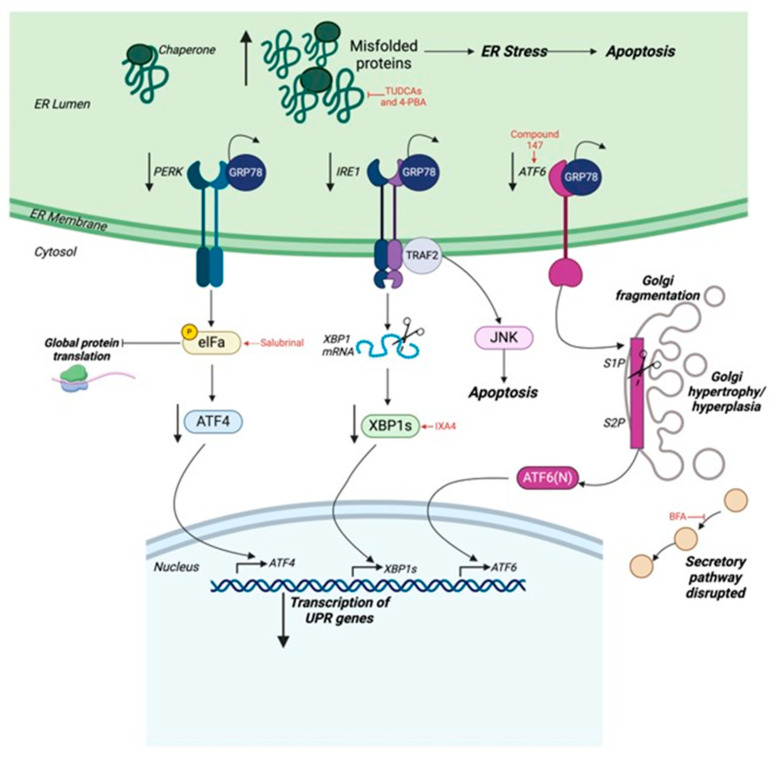
An illustration of maladaptive ER stress and Golgi dysfunction leading to heart failure. The three branches of the unfolded protein response (UPR) and their downstream molecular pathways are essential for resolving ER stress. A dysregulation of these pathways upon prolonged stresses contributes to the onset and progression of heart failure. Potential treatments are indicated in red. 4-PBA, 4-Phenylbutyric acid; ATF4, activating transcription factor 4; ATF6, activating transcription factor 6; BFA, Brefeldin A; eIFα, eukaryotic initiating factor α; ER, Endoplasmic reticulum; GRP78, glucose regulated protein-78; IRE1, inositol-requiring protein 1; JNK, Jun amino-terminal kinases; PERK, protein-kinase-like ER kinase; S1P, site 1 protease; S2P, site 2 protease; TRAF2, TNF receptor-associated factor 2; TUDCAs, Tauroursodeoxychloic acids; XBP1, X-box binding protein 1; XBP1s, spliced X-box binding protein 1.

Under physiological conditions, the UPR sensors are bound to the ER chaperone glucose-regulated protein-78 (GRP78) via their luminal domains. Once stimulated by the accumulation of misfolded proteins, they dissociate from GRP78, resulting in their activation by oligomerization and autophosphorylation. These UPR sensors may be activated at varying time points at different strengths [175]. Once activated, PERK phosphorylates eukaryotic initiating factor α (eIFα), which diminishes global protein synthesis by inhibiting the initiation of translation, thereby lowering the burden of newly synthesized proteins entering the ER. However, the translation of selective stress-responsive proteins, including C/EBP Homologous Protein, growth arrest, and DNA damage-inducible 34 (GADD34), GRP78, GRP94, and ATF4 is increased in an attempt to resolve ER stress (Figure 2) [173,176]. 

In addition, IRE1, upon dimerization and autophosphorylation, displays endoribonuclease activity, which is utilized for the splicing of X-box binding protein 1 (XBP1) mRNA. The newly formed spliced XBP1 (XBP1s) is a potent transcription factor that translocates to the nucleus and binds to the promoter regions of ER response elements I and II and mammalian UPR elements to regulate the expression of ER chaperones like GRP78 and calnexin that aid in protein folding. IRE1 also triggers the expression of ERAD proteins such as ER degradation-enhancing α-mannosidase-like proteins, ER oxidoreductin 1, and ribosome-associated membrane protein-4 to facilitate the removal of misfolded proteins by targeting them for degradation in the proteasome [172]. On the other hand, IRE1 co-operates with tumor necrosis factor (TNF) receptor-associated factor 2 to activate c-Jun amino-terminal kinases via the stimulation of apoptosis signal-regulating kinase 1 to induce apoptosis under chronic ER stress conditions (Figure 2) [177]. 

Finally, ATF6, which separates from GRP78 under ER stress, is transported to the Golgi apparatus (GA), where it is proteolyzed by the Golgi resident site 1 protease and site 2 protease. This results in the production of a soluble active fragment of ATF6 (ATF6(N)) that enters the nucleus to function as a transcription factor. ATF6(N) acts to promote the expression of proteins that help to alleviate ER stress, including ER chaperones, ERAD proteins, and pro-survival genes. ATF6 also induces the expression of lipid biosynthesis genes required for membrane formation, allowing for the expansion of the ER to accommodate protein folding demands (Figure 2) [178,179]. 

### 5.2. Adaptive and Maladaptive ER Stress During the Development of Heart Failure

HF is accompanied by multiple risk factors, including obesity, hyperglycemia, hypoxia, inflammation, and dyslipidemia, all of which trigger ER stress. Therefore, the activation of UPR, considered an adaptive ER stress, can tackle numerous pathological stresses. For instance, both isoproterenol-induced and pressure overload-induced hypertrophic and failing hearts exhibited increased levels of GRP78 and XBP1s, demonstrating the activation of UPR [180]. Similarly, elevated GRP78 was observed in DhaI salt-sensitive rats in response to high salt diet-induced hypertension [181]. Furthermore, cultures of primary neonatal rat ventricular myocytes exposed to ischemic conditions resulted in increased nuclear ATF6 expression, which was lowered upon reperfusion, highlighting the protective role of UPR in the heart upon hypoxic stress [182]. In addition, TAC-induced hypertrophic hearts displayed upregulated levels of the ER chaperones GRP94, GRP78, and calreticulin, indicating that UPR might aid in enhanced protein synthesis and folding triggered by cardiac hypertrophy [183]. The activation of the adaptive UPR pathway in response to stresses as above helps restore normal cellular function, which is crucial for heart health.

However, long-term pathological stress induces maladaptive ER stress, exacerbating the onset of HF in the absence of the protective effects of the UPR [184]. For example, cardiac-specific PERK knockout mice suffered from impaired cardiac function under chronic TAC [185], highlighting its role in protecting the heart from congestive HF under pressure overload. On the other hand, cardiac overexpression of PERK in a murine I/R model ameliorated myocardial injury by reducing the expression of pro-apoptotic factors [186], further emphasizing the cardio-protective role of PERK under stress. 

More evidence confirms the vital beneficial function XBP1s exert on cardiac function. A compelling 60% and 70% reduction in XBP1s and IRE1α levels, respectively, were uncovered in humans with dilated cardiomyopathy. In accordance, pressure-overloaded mice hearts stimulated with TAC displayed significantly suppressed XBP1s levels, suggesting that a loss of XBP1s might aggravate the progression of HF. This was evidenced by heightened cardiac dysfunction in mice models with cardiac XBP1s deficiency, which was rescued by overexpressing XBP1s in the heart [187]. Moreover, a reduction in myocardial XBP1s has been demonstrated in HFpEF mouse models, owing to diminished IRE1α phosphorylation caused by its nitrosylation [188]. Interestingly, cardiac overexpression of XBP1s in mice alleviates the HFpEF phenotype, coupled with decreased myocardial lipid accumulation [189]. Similarly, cardiomyocyte-specific IRE1 overexpression preserved cardiac function and reduced fibrotic area in response to pressure overload [190], highlighting the importance of maintaining proper ER stress responses. 

Furthermore, pre-clinical studies have demonstrated the protective role of ATF6 in the heart. For instance, following subjection to I/R injury, ATF6 knockout mouse hearts displayed larger infarct sizes and increased cell death, as well as blunted UPR activation. Interestingly, ATF6 overexpression was successful in rescuing these harmful phenotypes [191]. Adding to this, a study by Hofmann et al. has revealed that ATF6-deficient mice exhibited profound pathological cardiac remodeling in response to isoproterenol stimulation, higher misfolded protein accumulation, and reduced myocardial contractility under pressure overload stress [192]. 

### 5.3. Targeting ER Stress in Heart Failure

Enhancing the adaptive pathway of the UPR or inhibiting the destructive pro-apoptotic signaling caused by maladaptive ER stress could prove to be an effective therapeutic strategy for cardiovascular complications. Numerous pharmaceutical approaches have been studied for their potential beneficial effects in treating HF by alleviating ER stress. One such molecule is the naturally occurring tauroursodeoxychloic acids (TUDCAs) [193]. These are small chemical chaperones that reduce ER stress by lowering misfolded proteins and stabilizing their structure. Administration of TUDCAs prevented cardiomyocyte contractile dysfunction and Ca^2+^ defects induced by a high-fat diet in mice [194]. Moreover, in a murine model under pressure overload stress, oral administration of TUDCAs significantly attenuated pathological cardiac remodeling, indicated by hypertrophy, fibrosis, and apoptosis [195]. However, there have been no clinical applications in the setting of HF. 

A 4-Phenylbutyric acid (4-PBA) is another chemical chaperone that helps to reduce ER stress by preventing misfolded protein aggregation and promoting accurate protein folding [196]. A study by Luo et al. revealed that 4-PBA administration was successful in restricting ER stress and, consequently, TAC-induced cardiac hypertrophy and fibrosis in mice [197]. Furthermore, the beneficial effects of 4-PBA on I/R injury have been demonstrated: isolated rat hearts exposed to I/R exhibited improved cardiac function, accompanied by reduced ER stress and apoptosis levels [198]. The 4-PBA is a clinically approved drug that is utilized as a form of therapy in urea cycle disorders; however, its exact role in the heart, particularly in humans, is yet to be investigated [199]. 

Moreover, compound 147, a selective activator of ATF6, has been shown to be able to reduce the activation of fibroblasts; however, in-depth studies are required to elucidate its effect on cardiac function and therapeutic potential [200]. Similarly, the XBP1s-selective pharmacological activator, IXA4, has proven to be effective in improving glucose metabolism, liver insulin action, and overall pancreatic function in diet-induced obese mice [201], although, again, its effects on the heart are unexplored. 

An additional class of therapeutics targeting the ER is eIF2α phosphatase inhibitors, which can regulate protein synthesis, stress response pathways, and translation initiation by inhibiting the phosphatases that control eIF2α dephosphorylation [202]. Salubrinal is an example of an eIF2α phosphatase inhibitor that has been studied in HF. Administration of salubrinal diminished cardiac hypertrophy and fibrosis in mice under TAC, as well as in isolated neonatal rat ventricular cardiomyocytes [203]. In accordance, in a rat HF model induced by coronary artery ligation, treatment with salubrinal significantly improved cardiac function while lowering apoptosis levels, emphasizing its therapeutic potential [204]. In summary, the UPR is a protective response; therefore, a deeper understanding of the modulation of the UPR is required to adequately exploit its potential to treat HF (Figure 2). 

### 5.4. Golgi Apparatus in Heart Failure 

The GA is a highly dynamic organelle formed from a series of stacked membranes. The GA is comprised of a cis and a trans side with a tubular reticular membrane network and a medial area of disc-shaped flattened cisternae. The cis-Golgi network controls the receipt of cargo from the ER, whereas the trans-Golgi network is responsible for the exit of cargo out of the GA to other areas of the cell and out of the cell [205]. Another crucial role of the GA, like the ER, is the regulation of post-translational modification of proteins. Glycosylation, phosphorylation, sulfation, and proteolysis of proteins entering from the ER are among these modifications. Moreover, the GA acts to convert ceramides from the ER into sphingolipids, which are rich components of the plasma membrane in mammals, emphasizing its role in lipid metabolism as well [206]. 

Existing evidence reveals a relationship between dysfunction of the GA and its impacts on the cardiovascular system. Firstly, swelling of the GA has been linked to CVDs. For instance, in failing canine hearts, increased size and number of Golgi bodies have been observed [207], while hypertrophy and hyperplasia of the GA were discovered in severely and chronically mechanistically stressed hearts [208]. Moreover, a mutation in the PLEKHM gene, physiologically involved in endosomal trafficking, causes abnormal subcellular distribution of the GA, as well as endosomes, which was associated with the development of DCM [209]. Likewise, a study conducted on cardiac surgery patients revealed significant fragmentation of the GA in subjects with atrial fibrillation. This implies the role of GA remodeling in cardiovascular complications [210]. Due to its role in mediating protein trafficking, the preservation of Golgi homeostasis is crucial for ensuring the healthy function of the heart. Another study demonstrated that ER-Golgi transport can act as a regulator of hypertrophic growth in cardiomyocytes, as the overexpression of RAB1, which governs the transport of the transcription factor angiotensin II (AngII) receptor from ER to Golgi, enhancing cell growth in neonatal cardiomyocytes as well as pathological cardiac hypertrophy in mice [211,212]. Furthermore, the ability of the GA to secrete lipoproteins in the heart has been revealed, suggesting that impaired Golgi function may lead to toxic myocardial lipid accumulation owing to defective lipoprotein secretion [213]. In summary, previous studies highlight the intricate relationship between the GA and pathophysiological processes of the heart, ranging from structure, transport, and post-translational modification of proteins. However, more in-depth research is required to fully understand the involvement and pathophysiological function of the GA in the heart (Figure 2). 

Given the possible link between impaired GA and HF, aspects of the GA could potentially be utilized as attractive targets for therapeutics against heart diseases; however, a very limited volume of research has been conducted in this area. Nonetheless, recent studies have shed light on numerous potential drugs that can target features of the GA in various diseases. For instance, compounds targeting GA-induced cytotoxicity, such as LTX-401, have been shown to be effective in anti-cancer therapies [214]. Similarly, molecules influencing GA-associated transport, including inhibitors of the Golgi-associated lipid transfer proteins such as ceramide transfer protein and oxysterol-binding protein and inhibitors of ARF activation like Brefeldin A, have also been tested for their therapeutic potential [215]. Notably, however, none of the above have been tested in the heart; therefore, their effectiveness against cardiovascular complications remains ambiguous. This highlights the need for extensive studies on such drugs to determine potential clinical applications. 

## 6. Autophagy

### 6.1. Autophagy in Cardiac Physiology

Autophagy is a tightly regulated cellular degradation process in which portions of the cytosol and damaged or unnecessary organelles are broken down and recycled. Autophagy can be categorized into three primary types: microautophagy, macroautophagy, and chaperone-mediated autophagy. While they share a common goal of lysosome degradation, each pathway presents different mechanisms [216,217]. 

A family of autophagy-related genes (ATG) is crucial for regulating and carrying out autophagy [218]. Functionally, human ATG proteins are categorized into four main groups: (1) the unc-51-like kinase (ULK) complex is central to the initiation of autophagy; (2) two ubiquitin-like (UBL) conjugation systems [219]. The first system facilitates the formation of the ATG12–ATG5-ATG16 complex, which acts as a scaffold to recruit and stabilize essential components at the phagophore [220]. The second system involves the lipidation of the autophagosome marker LC3, a critical step for elongating and closing the autophagosomal membrane; (3) two ER transmembrane proteins, ATG9 and vacuole membrane protein 1, crucial for membrane trafficking and remodeling during autophagosome formation; (4) a class III phosphatidylinositol 3-kinase (PI3K-III) complex, catalyzing the phosphorylation of phosphatidylinositols (PtdIns) to yield phosphatidylinositol 3–phosphate (PtdIns3P) thereby facilitating membrane dynamics and trafficking essential for autophagosome formation and maturation [221]. 

Mammalian homologs of ATG1, including ULK1 and ULK2, play a key role in autophagy activation and work downstream of nutrient-sensing pathways such as the mTORC1 pathway. Under nutrient-rich conditions, mTORC1 inhibits autophagy by phosphorylating ULK1 at Ser758 [222]. When mTOR is inhibited, the suppression is lifted, allowing ULK1 to become susceptible to activation. During starvation, the ULK1 complex is further activated by AMPK through phosphorylation at Ser317 and Ser777, promoting the initiation of autophagy [222,223]. Once activated, the ULK1 complex drives the formation of the phagophore through two pivotal mechanisms. First, it activates the PI3K-III complex comprising Beclin 1, vacuolar protein sorting 34 (VPS34), VPS15, and ATG14-like (ATG14L). This generates PtdIns3P, which facilitates the recruitment of autophagy-related proteins to the phagophore assembly site on membranes [224]. Secondly, ULK1 regulates the trafficking of ATG9 to promote the delivery of membrane material to the expanding phagophore, ensuring its elongation and closure. Afterward, the sealed autophagosome fuses with a lysosome, where the sequestered material is degraded by lysosomal hydrolases within the resulting digestive autolysosome, and the breakdown products are exported back into the cytoplasm for reuse by the cell [225]. 

### 6.2. The Dual Role of Autophagy in Heart Failure 

Physiologically, enhanced autophagic activity is an adaptive response to reduce cellular stress and preserves cardiomyocyte integrity under increased workload. This upregulated autophagy supports cellular survival by providing energy and maintaining homeostasis during periods of heightened stress [226]. Impaired autophagy contributes to various CVDs, including HF [139,227]. In HF, patient biopsies reveal downregulation of autophagy-related proteins, such as Beclin1 and LC3II, indicating reduced autophagosome formation and, thus, autophagy [228]. Cardiac-specific deletion of Atg5 resulted in LV dilation and dysfunction one week post-TAC [226]. Cardiac tissue from ATG5 knockout mice showed disrupted sarcomere arrangement and damaged mitochondria [229]. 

Moreover, Zhang et al.’s study describes a pathway by which impaired autophagy drives cardiac dysfunction, initiated by the accumulation of the protein p62. This accumulation activates NF-κB, which then translocates to the nucleus and upregulates the expression of nicotinamide N-methyltransferase, ultimately contributing to cardiac dysfunction [230]. 

Lysosomal function is an integral part of the autophagic process, and defects in this consequently lead to a reduction in autophagy. A number of studies have indicated a link between lysosomal storage diseases and CVDs, including HF [231,232]. Humans with amyloidogenic HF exhibit dysregulated autophagy, and further experimentation has revealed lysosomal dysfunction as a significant cause for this [233]. Furthermore, in the hearts of mice subjected to TAC, it has been observed that there is a significant decrease in autophagy-associated lysosome expression. This correlated with the accumulation of damaged organelles due to insufficient autophagy [234]. The lysosomal-associated membrane protein 2 (LAMP2) is a protein essential for autophagosome-lysosome fusion, and it is vital for autophagy to proceed in cardiomyocytes [235]. In LAMP2-deficient aged mice, HF progression is exacerbated alongside a reduction in lysosome biogenesis and, thus, autophagy [236]. 

Insulin is a key hormone required for regulating cardiac metabolism, and impairments in its signaling pathways have been associated with HF in part due to its ability to inhibit autophagy [237]. Cancer patients and mice presenting with either B16F10 melanoma or C26 carcinoma displayed similar decreases in serum insulin levels. Furthermore, these cancerous mice developed HF, which was attenuated by insulin supplementation. It appeared that this, in part, arose due to an insulin-induced decrease in autophagy, as exhibited by a reduction in the expression of autophagic proteins [238].

When overactivated, autophagy can shift from being protective to contributing to cardiac pathology. Hypertrophic stimuli can lead to prolonged activation of myocardial autophagy, causing abnormalities and dysfunction of intracellular structures [239,240]. Inhibiting autophagy by beclin 1 haploinsufficiency or histone deacetylases (HDACs) inhibitor trichostatin A, which inhibits autophagy, has been shown to reduce cardiac hypertrophy and prevent progressive cardiac dysfunction [241,242]. These results suggest that sustained autophagy activation is key in driving the progression from cardiac hypertrophy to HF. 

### 6.3. Targeting Autophagy in Heart Failure

Autophagy plays a complex yet vital role in the development of HF, and multiple drugs targeting these mechanisms have been identified as potentially beneficial in the treatment of HF. The Mayo Clinic, USA, has commenced trials to assess the efficacy of the mTORC1 inhibitor rapamycin for the treatment of HF. In rats, rapamycin treatment conferred a degree of protection against MI-induced systolic dysfunction via promoting autophagy and inhibiting apoptosis [243]. Targeting beclin-1, as a key component of autophagy progression, could also be beneficial for the treatment of HF. In sepsis-induced HF, treatment with the peptide Tat-beclin-1 reduced cardiotoxicity and improved cardiac function via increasing autophagy [244]. However, the efficacy of targeting beclin-1 in humans with HF is yet to be investigated. Conversely, inhibition of PI3K via the compound 3-MA has been used to suppress doxorubicin-induced excess autophagy and to improve the associated systolic dysfunction and structural remodeling [245]. This highlights the importance of fine-tuned modulation of autophagy as a therapeutic strategy. 

Improving lysosomal biogenesis and function offers a potential therapeutic avenue for the treatment of HF. Indeed, cilostazol, a drug primarily employed to reduce platelet aggregation due to its role as a phosphodiesterase 3 inhibitor, has been shown to ameliorate cardiac dysfunction and reduce N-terminal pro-B-type natriuretic peptide (NT-proBNP) levels in patients with HFpEF [246]. An additional study in rats revealed that cilostazol not only improved I/R-induced apoptosis but increased autophagy via enhanced nuclear expression of transcription factor EB (TFEB). TFEB is a key regulator of many proteins involved in lysosomal biogenesis and function, and cilostazol treatment consequently increases the expression of LAMP1 and LAMP2 [247]. 

### 6.4. Impaired Mitophagy in Heart Failure

Mitophagy is a quality control mechanism to remove damaged mitochondria through autophagy, playing an essential role in preserving mitochondrial function in the cardiomyocytes. Under physiological conditions, basal mitophagy is continuously activated to sustain mitochondrial recycling and turnover, maintaining energetic homeostasis and reducing oxidative stress. While some components of the mitophagic pathway are shared with autophagy, there is a selection of proteins of great importance in mediating mitophagy. PINK1 acts as a sensor and marker for mitochondrial damage, and its recruitment at the mitochondrial membrane initiates mitophagy. The ubiquitin ligase Parkin is activated by PINK1 and labels the mitochondria as ready for degradation [248]. Maintaining PINK1 and Parkin levels is essential for the clearance of damaged mitochondria in response to stress, and their deletion exacerbates high-fat-induced cardiac dysfunction, reduces post-MI survival, and increases the rate of pathological structural cardiac remodeling [249,250,251]. However, in obesity [250] and post-MI [252], endogenous Parkin expression is significantly reduced, indicating a decrease in mitophagic capacity.

Several pharmacological approaches aim to activate mitophagy to delay HF progression. Recently, Nuanxinkang has been reported to enhance Parkin-related mitophagy, preventing heart remodeling in a mouse model of HF [253]. Similarly, omentin1 overexpression, induced by fat restriction, upregulated Parkin-mediated mitophagy and improved cardiac function in mice with myocardial ischemia-induced HF [254]. Additionally, evidence also indicates that empagliflozin, an FDA-approved antidiabetic drug, activates mitophagy, promoting mitochondrial turnover, improving cardiomyocyte energy metabolism, and reducing adverse LV remodeling in HF models [255]. Berberine is a compound found in the Chinese herb coptis, and its use as a pro-mitophagic drug for the treatment of HF has been investigated. In mice, berberine alleviated TAC-induced cardiac dysfunction and structural remodeling, while its cardioprotective effects were abolished when PINK1 was downregulated, indicating that it acts to restore TAC-induced decrease in mitophagy [252]. Tat-Beclin1 has been found to activate mitophagy, attenuate mitochondrial dysfunction, reduce lipid accumulation, and protect against mitochondrial dysfunction and cardiac dysfunction in mice models with HF [256].

## 7. Inflammation

### 7.1. Pro-Inflammatory Cytokines in the Heart

Pro-inflammatory cytokines play a pivotal function in cell signaling within the heart, shaping both physiological and pathological processes. The cytokines facilitate tissue repair and remodeling by enhancing angiogenesis and catalyzing the proliferation of cardiac fibroblasts, which are intrinsic in maintaining the structural integrity of the heart [257]. The roles of the proinflammatory cytokines interleukin (IL)-1β, IL-6, and TNFα in HF have been extensively studied, with vast quantities of the literature available, indicating a positive correlation between themselves and disease progression [258,259,260,261]. Of note, these cytokines provide an additional link between cancer and HF development due to crosstalk between tumor microenvironments and the myocardium [8]. While we cannot neglect the importance of these inflammatory factors, an advanced understanding of new mediators is necessary to explore the inflammation-involved pathogenesis of HF.

#### 7.1.1. HMGB1

**Figure 3:**
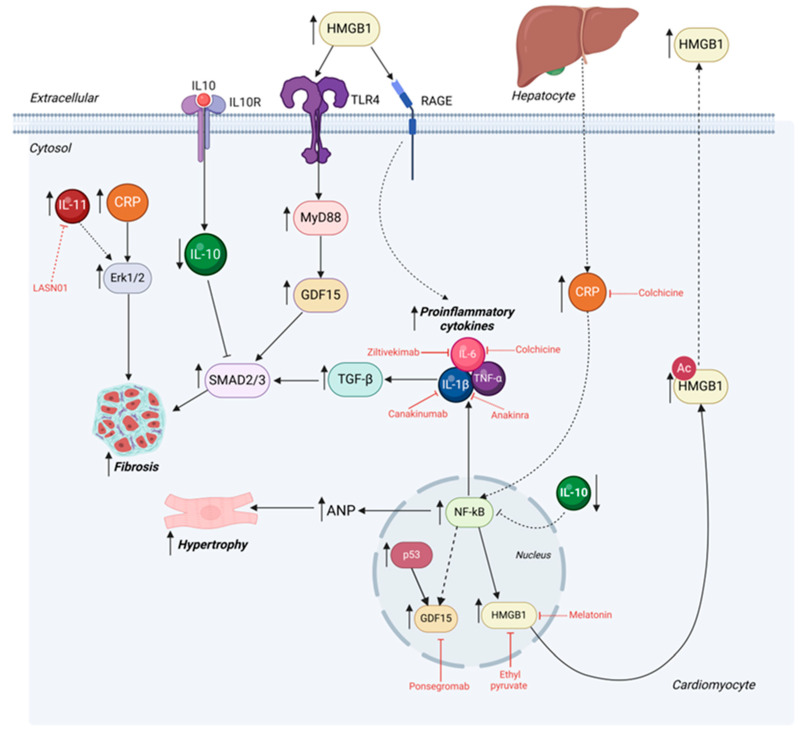
A diagram of dysregulated inflammatory mechanisms in heart failure. In physiological conditions, a fine equilibrium between pro and anti-inflammatory markers is vital for proper cardiac function. However, in the diseased state, this is disrupted, resulting in enhanced pro-inflammatory molecules, such as HMGB1 and GDF15, and decreased anti-inflammatory molecules, such as IL-10, which leads to higher levels of IL-1β, TNF-α, and other cytokines, triggering pathological cardiac remodeling. Potential treatments are indicated in red. ANP, Atrial natriuretic peptide; CRP, C-reactive protein; ERK, Extracellular signal-regulated kinase; GDF15, Growth differentiation factor 15; HMGB1, High mobility group box 1; IL, Interleukin; MYD88, Myeloid differentiation primary response 88; NF-kB, Nuclear factor κB; RAGE, Receptor for advanced glycation endproducts; SMAD, suppressor of mothers against decapentaplegic; TGF-β, Transforming growth factor β; TLR, Toll-like receptor; TNF-α, Tumor necrosis factor α.

High mobility group box 1 (HMGB1) is a non-histone protein that, under physiological conditions, resides within the nucleus, acting as a DNA chaperone critical for chromosomal conformation and transcriptional regulation [262]. In HF, the activation of HMGB1 occurs through distinct pathways, each facilitating the role of HMGB1 as a critical inflammatory modulator following its active or passive release (Figure 3) [262].

The active secretion of HMGB1 entails a complex series of post-translational modifications. The initial step is the acetylation of specific lysine residues within the nuclear localization sequence to prevent the nuclear re-entry of HMGB1 and facilitate its cytoplasmic accumulation [263]. This process is modulated by histone acetyltransferases and HDACs, such as CREB-binding protein and SIRT1, respectively, in response to inflammatory signals induced by NF-κB [263]. Additionally, phosphorylation by Ca^2+^-dependent PKC further amplifies the cytoplasmic translocation of HMGB1 [263]. Activated macrophages and monocytes actively release HMGB1 via a non-classical vesicular pathway in response to mechanical and oxidative stress [263]. Passive release of HMGB1 occurs during necrosis, a prominent feature of HF. Once cardiomyocytes undergo necrotic cell death due to mechanical stress or oxidative stress, an unregulated release of intracellular content occurs, including HMGB1. The passive secretion alerts the immune system to tissue damage and initiates the inflammatory response [263]. 

Oxidative stress promotes the oxidation of critical cysteine residues within HMGB1, which affects its release and the subsequent inflammatory response. In addition, elevated cardiac wall tension and abnormal mechanical loading trigger the translocation of HMGB1 from the nucleus to the extracellular space, where HMGB1 can bind to toll-like receptor 4 (TLR4) and receptor for advanced glycation end products to initiate inflammatory cascades and increase pro-inflammatory cytokines, such as TNFα. This subsequently elevates pro-fibrotic mediators [262], such as transforming growth factor β (TGF-β), and cell growth factors, such as atrial natriuretic peptide, leading to cardiac hypertrophy and, ultimately, HF.

In patients with acute MI, researchers have observed markedly increased levels of HMGB1 in the blood, peaking 12 h after the infarction event. Elevated levels of HMGB1 were correlated with detrimental outcomes, including in-hospital cardiac mortality, cardiac rupture, and pump failure [264]. Moreover, studies have found a direct association between elevated levels of HMGB1 in the blood, which indicates that overexpressing HMGB1 aggravates poorer outcomes in MI patients by enhancing inflammatory responses [265]. In addition, TLR4-mutant mice displayed diminished myocardial apoptosis and an overall reduction in myocardial injury under I/R conditions [266]. Conversely, overexpressing HMGB1 enhanced cardiac apoptosis and inflammatory responses [267]. 

#### 7.1.2. GDF15

The transcriptional activation of GDF15 emerges mainly via p53-dependent mechanisms in HF [268]. Additionally, pro-inflammatory cytokines can further induce transcription of GDF15. The activation of TLRs, such as TLR4, by danger-associated molecular patterns (DAMPs) can induce the expression of GDF15 via the MyD88 signaling mechanism (Figure 3) [268]. 

Cardiac mechanical strain can stimulate the expression of GDF15, which becomes significant in pathological cardiac remodeling and HF. Post-transcriptional modulation of GDF15 entails the proteolytic cleavage of its N-terminal pro-peptide by furin-like proteases in the secretory pathway, resulting in the release of the active dimeric form of GDF15 [269,270]. Furthermore, GDF15 induces the transformation of fibroblasts to myofibroblasts via a suppressor of mothers against decapentaplegic (SMAD)-dependent signaling pathway. The activated myofibroblasts can enhance the production of extracellular matrix proteins, such as collagen types I and III, which contribute to interstitial fibrosis and modified cardiac mechanics. Moreover, GDF15 can regulate inflammatory cell recruitment and activation via interactions with its receptor GDNF family receptor α-like [271,272].

In Biology Study to Tailored Treatment in Chronic Heart Failure, it was unveiled that in HF, elevated levels of GDF15 were correlated with reduced protein intake and lower urinary creatinine, which indicates muscle wasting [273]. This has identified GDF15 as an important predictor of mortality in HF. In GADD34 knockout mice, radiation exposure resulted in dilated cardiomyopathy and severe weight loss. GADD34 indirectly negatively regulates GDF15 expression; thus, the level of GDF15 in these mice was elevated in cardiomyocytes and circulation [273]. Once treated with GDF15-blocking antibodies, the mice were able to maintain body weight and exhibited a slower progression of HF. This indicates that GDF15 has an influential role in cardiac cachexia and HF development [273]. 

#### 7.1.3. CRP

C-reactive protein (CRP) is an acute-phase protein synthesized by the liver, which serves as a marker of inflammation correlating with cardiovascular morbidity and mortality [274]. It is induced by IL-6 binding to IL-6 receptor α chain and Janus kinases/signal transducer and activator of transcription proteins (JAK/STAT) signal transduction [274]. Additional pro-inflammatory cytokines amplify the expression of CRP through NF-κB-dependent pathways [275]. Oxidative stress modulates the expression of CRP via redox-sensitive transcription factors. The Keap1-NRF2-ARE pathway, through its control over ROS, indirectly regulates CRP transcription. 

CRP induces the differentiation of cardiac fibroblasts to myofibroblasts via ERK1/2 and PI3K/Akt signaling pathways. Also, CRP enhances inflammatory cytokine expression through NF-κB-dependent mechanisms in cardiomyocytes, facilitating the infiltration of macrophages, which, in turn, enhances the expression of CRP in a vicious cycle that worsens cardiac remodeling and function, leading to HF (Figure 3) [276]. However, further validation of this pathway in cardiac fibrosis is needed to confirm the relationship. 

The Utrecht Cardiovascular Cohort-Second Manifestations of Arterial Disease study examined around 9000 patients over a period of almost 10 years [277]. They found that elevated CRP levels independently predicted HF development [277]. They indicated several pathways through which CRP can contribute to HF, including the promotion of the inflammatory response, which leads to dysfunctional myocardial contractility via profibrotic tissue formation [277]. 

#### 7.1.4. IL-11

IL-11 is a member of the IL-6 cytokine family and is implicated in positively regulating myocardial fibrosis [278]. Transcriptional activation of IL-11 is modulated through a TGF-β-dependent pathway and SMAD2/3 phosphorylation. Also, increased mechanical load activates focal adhesion kinases and the subsequent ERK1/2 pathway, resulting in augmented IL-11 transcription [279]. Moreover, NF-κB-dependent mechanisms increase the expression of IL-11 following inflammatory stimuli. Additionally, oxidative stress augments the expression of IL-11 via redox-sensitive transcription factors, such as NF-κB. 

IL-11 activates the ERK1/2 pathway, which leads to the augmentation of pro-fibrotic genes, such as fibronectin, culminating in cardiac interstitial and perivascular fibrosis [279]. However, this pathway needs further validation. In addition, IL-11 impairs Ca^2+^ handling in cardiomyocytes via the glycoprotein 130 receptor complex. This will lead to transcriptional degradation of Ca^2+^ handling proteins, such as sarcoplasmic reticulum Ca^2+^-ATPase 2a (SERCA2a), the primary protein responsible for Ca^2+^ reuptake into the sarcoplasmic reticulum (SR) during diastole, resulting in compromised contractile function of the heart [280]. 

In HF patients, researchers have confirmed elevated levels of IL-11 [281]. This demonstrated that increased levels of IL-11 are associated with cardiac events in HF, which indicates a potential role for IL-11 as a biomarker for cardiac dysfunction [281]. It has been demonstrated that direct injection of recombinant IL-11 induced cardiac fibrosis in wild-type mice [278]. This effect was diminished in IL-11 receptor knockout mice, which illustrated the requirement of IL-11 signaling for fibrotic tissue formation [282]. Moreover, in transgenic mice, fibroblast-specific overexpression of IL-11 led to cardiac fibrosis, reinforcing the pro-fibrotic effects of IL-11 [281].

### 7.2. Macrophage Infiltration in the Heart

The infiltration of macrophages in the heart arises via a complex cascade of events that are initiated by cardiac injury or stress. Following MI, necrotic cardiomyocytes release DAMPs, including HMGB1, ATP, and mitochondrial DNA. The DAMPs bind to pattern recognition receptors (PRRs), such as TLR4, on cardiac cells to initiate the inflammatory response. The activated cell releases chemokines, such as CCL2 and CCL7, which recruit monocytes from the circulation. In the meantime, endothelial cells elevate adhesion molecules, such as intercellular and vascular cell adhesion molecules-1 (ICAM-1 and VCAM-1), to facilitate monocyte adhesion and transmigration into cardiomyocytes [283]. 

Infiltrated monocytes differentiate into macrophages due to cytokine modulations, such as granulocyte-macrophage- and macrophage-colony-stimulating factors. Depending on the initiated signal, macrophages exhibit distinct phenotypes as either pro-inflammatory M1 macrophages or M2 macrophages associated with tissue repair and fibrosis [284]. The sustained mechanical stress and oxidative stress preserve the recruitment signal; thereafter, this prolonged recruitment maintains a vicious cycle where infiltrating macrophages release more pro-inflammatory modulators, recruiting additional inflammatory cells and augmenting the inflammatory response [285]. This exacerbates cardiac remodeling and cardiac dysfunction, ultimately leading to HF. 

### 7.3. Anti-Inflammatory Cytokines in the Heart 

The main role of anti-inflammatory cytokines is to resolve inflammation and prevent excessive tissue damage. It is initiated via specific molecular pathways triggered by the inflammatory response itself, which represents a critical homeostatic mechanism. Cardiac-resident macrophages induce IL-10 and TGF-β following the phagocytosis of apoptotic cardiomyocytes. This activates STAT3-dependent transcription of anti-inflammatory modulators [286]. 

Once IL-10 binds to its receptors on cardiomyocytes, it will augment the expression of anti-apoptotic proteins, significantly downregulating cardiomyocyte death [286]. Moreover, IL-10 suppresses pro-inflammatory cytokine production via the inhibition of NF-κB-dependent pathways in cardiomyocytes. In addition, it attenuates the expression of adhesion molecules on cardiac endothelial cells, limiting the infiltration of inflammatory cells [287]. IL-10 culminates in altered extracellular matrix homeostasis, limiting the adverse cardiac remodeling. It also reduces the differentiation of fibroblasts into myofibroblasts, thereby reducing fibrotic tissue formation [288]. Therefore, it is unsurprising that in humans with chronic HF, serum IL-10 levels were significantly reduced [289]. Further studies on rats subjected to MI confirmed the role of IL-10 in HF development as there was a continuous decrease in the mRNA expression of IL-10 and the concentration of membrane-bound IL-10 post-MI correlating with an increase in proinflammatory cytokine TNFα [290].

### 7.4. Targeting Myocardial Inflammation in Heart Failure 

Intriguingly, despite being an infamous marker for cardiac inflammation in HF, therapeutic targeting of TNFα has proven to be somewhat unsuccessful. Administration of the TNFα monoclonal antibody infliximab to patients with HFrEF had disastrous consequences, with there being no improvement and a significant increase in the risk of death in those receiving a low or high dose of the drug, respectively, despite a decrease in proinflammatory markers [291]. Hence, the complex interplay between multiple inflammatory modulators requires a strategic combination of approaches targeting different inflammatory pathways to achieve therapeutic efficacy.

Ethyl pyruvate, a lipophilic pyruvate derivative, has demonstrated potential as a primary HMGB1 inhibitor. Studies have shown that it can reduce TNFα and IL-6 levels while preserving myocardial function via disruption in HMGB1-cytokine binding [292]. Melatonin, a naturally endogenously derived circadian indoleamine, reduced inflammation and recovered cardiac function following MI via regulating the miR-200b-3p-HMGB1 pathway [293]. Melatonin has completed phase 2 trials, showing positive effects in regard to reducing complications following acute MI [294]. 

Furthermore, researchers have demonstrated that blocking the activity of GDF15 via a monoclonal antibody had significant therapeutic potential [273]. A phase 2 randomized clinical trial is actively assessing ponsegromab, a monoclonal anti-GDF15-neutralizing antibody (NCT05492500), and its efficacy in treating HF in patients who exhibited upregulated levels of GDF15. 

ILs can be targeted for the treatment of HF, and this therapeutic approach was investigated in the Canakinumab anti-inflammatory thrombosis outcome study trial. Researchers revealed that treatment with canakinumab, an anti-inflammatory antibody that targets IL-1β, decreased CRP levels and reduced the risk of cardiovascular events [295]. Further to this, anakinra is a drug that inhibits IL-1 receptors, thereby ameliorating IL-1-induced inflammation. The Recently Decompensated Heart Failure Anakinra Response Trail involving patients with acute HF revealed that those treated with anakinra for 12 weeks exhibited an improvement in LV filling pressure, indicating its potential as a therapy for individuals with this disease [296]. Currently, a phase 2 clinical trial is evaluating a specific antibody, called LASN01, that targets IL-11 receptor α, which has shown promising results in pre-clinical bleomycin models of fibrosis (NCT05331300) (Figure 3). Finally, the potentially groundbreaking HERMES trial is currently underway to determine the efficacy of the monoclonal antibody ziltivekimab, targeting IL-6, in patients with HFpEF and HFmrEF, although no data are available yet.

In addition, there are multiple clinical trials currently ongoing investigating the use of anti-inflammatory drugs for the treatment of acute HF, although data from these have not yet been released [297].

A significant problem with targeting inflammation is the fact that there is a huge variation in the inflammatory profile between individuals with HF regardless of subtype, rendering generic anti-inflammatory drugs inappropriate. Since the introduction of CART in oncology, there has been great interest in the development of therapies designed regarding the specific molecular and genetic profiles of an individual. Macrophage polarization as a disease target offers significant potential for personalized treatment of HF [298]. While not in the heart, autologous M2 macrophage transplantation improved cognitive function in children with cerebral palsy with no adverse effects, highlighting the prospective benefits of taking such a novel therapeutic approach [299]. Furthermore, in a clinical trial of patients with HFrEF, transendocardial injection of ixmyelocel-T, a cellular therapy consisting of CD90^+^ mesenchymal stem cells, and CD45^+^CD14^+^ macrophages derived from an individual’s own bone marrow exhibited a significant decrease in cardiovascular events [300].

## 8. Programmed Cell Death

### 8.1. Apoptosis in Cardiac Physiology

Apoptosis is a form of programmed cell death arising in response to specific danger signals indicative of a harmful and dysfunctional intra- and extracellular environment. A key feature of HF, particularly HFrEF, is the loss of cardiomyocytes via apoptosis, contributing to a significant loss in the amount of contractile myocardial tissue, thereby exacerbating disease development [10]. 

The intrinsic pathway is mediated by the BCL2 family of proteins and is induced by danger signals originating from within the cell, including mitochondrial dysfunction, ROS, and DNA damage. In response, proteins BH3, interacting-domain death agonist, BCL-2-like protein 11, and p53 upregulated modulator of apoptosis (PUMA) activate mitochondrial membrane proteins BCL-2-like protein 4 (BAX) and BCL2 Antagonist/Killer 1, triggering their oligomerization for pore formation and mitochondrial outer membrane permeabilization, considered as the stage at which a cell is committed to death. Cytochrome c is released into the cytosol by the damaged mitochondria, initiating the formation of the apoptosome and activation of caspase-9. Caspase-9 cleaves and activates executioner caspases-3, -6, and -7, which deconstruct the cell [301]. 

External danger signals TNFα and FasL bind to their respective receptors, TNFR and FasR, to initiate the extrinsic pathway. TNFα binding triggers the recruitment of TNFR1-associated death domain protein, which further sequesters Fas-associated death domain protein (FADD), while FasL binding directly causes FADD appropriation. FADD associates with and activates the executioner caspase-8 [302]. This pathway is negatively regulated by the FLICE-inhibitory protein (FLIP), which can bind to and inhibit both FADD and caspase 8 [303].

### 8.2. Apoptosis in Heart Failure

In failing human hearts, increased activated caspase-9, caspase-3, and cytosolic cytochrome c have been observed, suggesting that there was increased apoptosis [304,305]. Additionally, pro-apoptotic proteins, including BAD, PUMA, and BAX, are elevated, while anti-apoptotic proteins BCL-2 and BCL-XL are decreased in various models of HF. There is also a significant increase in p53 expression in humans with HF [306,307], with similar observations having been made in vivo models [308].

Runt-related transcription factor 1 (RUNX1) is a transcriptional regulator of p53, and its expression is elevated in HF, as observed in mice subjected to TAC [309]. In addition to a reduction in the proportion of TAC-induced apoptotic cells, following the knockdown of RUNX1, the expression of p53 was similarly decreased, as well as that of BAX and PUMA [309,310]. Subsequent to I/R injury, nuclear translocation of p53 requires the binding protein apoptosis stimulating of p53 protein 1 (ASPP1). Expression of ASPP1 and p53 in the nuclear fraction is increased following I/R, and, in the absence of ASPP1, p53 is unable to translocate, and thus, the I/R-induced apoptosis is alleviated [311]. 

As aforementioned, TNFα levels are significantly increased in HF, and it is confirmed that FasL, a membrane protein located on CD8+ T cells, is also elevated in the failing heart [312]. Caspase-8 is increased in the hearts of HF patients, correlating with a decrease in anti-apoptotic protein FLIP [305]. A separate clinical study found a positive correlation between caspase-8 expression and the incidence of coronary events [313]. 

Receptor-interacting protein kinase 1 (RIPK1) plays a significant role in mediating the formation of the caspase-8/FADD complex, and modulation of RIPK1 regulatory mechanisms can improve HF-induced apoptosis. In both pressure overload and infarction murine models, TGF-β-activated kinase 1 binding protein 2 (TAB2) was reduced in correlation with cardiac dysfunction. Deletion of myocardial TAB2 contributes to an increase in RIPK1 expression and, therefore, apoptosis. Further in vitro analysis confirmed that TAB2 deficiency results in activation of the extrinsic apoptosis pathway [314]. Finally, HF was correlated with a decrease in the extrinsic pathway inhibitor FLIP [315]. In a hypoxia/reperfusion model, overexpression of FLIP decreased apoptosis and had the additional benefit of reducing ER stress via inactivation of the p38 MAPK pathway [316]. 

### 8.3. Targeting Apoptosis in Heart Failure

A major hurdle in the development of anti-apoptotic drugs is that for many years, much focus has been on triggering apoptosis induction for the treatment of cancers. However, in vivo, the inhibition of p53 presents potential as an approach for treating HF. In both mice with doxorubicin-induced HF and a rat model of I/R injury, administration of p53 inhibitor pifithrin-α decreased BAX expression and the proportion of apoptotic cells [317,318]. However, the expression of p53 itself remained unchanged, suggesting that this drug inhibits the transcriptional activity of p53. Caspase inhibition is another therapeutic approach in treating HF. In an ex vivo rat model of I/R injury, the infarct percentage was significantly reduced in receiving a non-selective caspase inhibitor Z-VAD-fmk as well as inhibitors specific for caspases-8, -9, and -3, named Z-IETD-fmk, Z-LEHD-fmk, and Ac-DEVD-cmk, respectively [319].

It may seem somewhat contradictory, but preclinical data suggest that the inhibition of anti-apoptotic proteins BCL-XL and BCL-2 can also improve outcomes in HF. Due to aging, the accumulation of senescent cells can lead to the development of a proinflammatory and profibrotic state. Navitoclax is one such drug, and its administration in AngII stressed, and doxorubicin-treated mice increased caspase-3 expression, correlating with a reduction in cardiac dysfunction, hypertrophy, and fibrosis [320,321]. Of course, one of the major drawbacks of this technique could be non-specific inhibition of the anti-apoptotic proteins, leading to excessive apoptosis.

### 8.4. Ferroptosis in Cardiac Physiology

Ferroptosis is a form of iron-dependent programmed cell death characterized by the accumulation of intracellular lipid peroxides and redox imbalance. In brief, intracellular ferrous iron (Fe^2+^) interacts with cellular H_2_O_2_ to form hydroxyl and hydroperoxyl radicals, which consequently cause FAO, particularly polyunsaturated FAs (PUFAs). The resulting lipid peroxides contribute to cell death via destruction of phospholipid membranes. 

Ferric iron (Fe^3+^) is transported from the bloodstream, reduced into Fe^2+^, and endocytosed into the cytosol of cardiomyocytes. Here, the Fe^2+^ can be utilized, stored by ferritin, exported, or remain unbound in the cytosol as redox-active labile iron. In conditions of high iron demand or iron depletion, Fe^2+^ is released from storage through ferritonophagy, which is mediated by nuclear receptor coactivator 4 (NCOA4) [322]. Intracellularly, there are two principal opposing mechanisms regulating ferroptosis. GPX4 utilizes GSH in a chemical reaction to convert H_2_O_2_ into H_2_O and is, therefore, essential for reducing the rate of PUFA oxidation in ferroptosis. Cysteine importation is the rate-limiting step in the synthesis of GSH, and the cysteine/glutamate antiporter xCT mediates this process [322]. Conversely, Acyl-CoA synthetase long-chain family member 4 (ACSL4) is an enzyme pivotal for ferroptosis mediation that catalyzes the esterification of CoA into PUFAs, triggering the process of phospholipid peroxidation [323]. Finally, NRF2 is considered a master transcriptional regulator, which is responsible for the transcription of anti-ferroptotic genes [322].

### 8.5. Ferroptosis in Heart Failure

Several human and in vivo studies have revealed that there was a decrease in GPX4 and an increase in oxidative stress in HF indicative of increased lipid peroxidation and, thus, ferroptosis. This hypothesis is further supported by the expression of proteins pivotal for iron metabolism being dysregulated and Fe^2+^ levels being elevated [324,325,326,327]. Regarding ferritonophagy, ferritin expression was increased, and the proportion of Fe^2+^ and Fe^3+^ were significantly elevated and decreased, respectively, in wild-type mice in response to TAC in comparison to NCOA4 KO mice [328]. This indicates that in HF, there is a significant increase in NCOA4-mediated ferritonophagy, resulting in elevated levels of labile iron. 

Ferroptosis regulatory proteins are dysregulated in HF. In AngII-treated mice, xCT levels were found to be initially reduced following exposure to hypertensive stress, and the ablation of xCT further aggravated cardiac hypertrophy in a ferroptosis-dependent manner [329]. Conversely, in mice subjected to TAC, ASCL4 expression was significantly increased and correlated with decreased GPX4 [330]. Hence, it is evident that in HF, the decrease and increase in the expression of the major anti- and pro-ferroptotic proteins, respectively, play a significant role in the observed elevated rate of ferroptosis. 

NRF2 expression is significantly decreased in failing human hearts due to the changes in ferroptotic mechanisms. The elevated expression of glycosylated matrix metalloproteinase-9 (MMP9) has a dual role in ferroptosis regulation. Firstly, it suppresses NRF2-mediated transcription, thus resulting in reduced expression of anti-ferroptotic genes. Secondly, MMP9 directly interacts with GPX4 and inhibits its activity [331]. Increased MMP9 levels were correlated with greater LV dysfunction following MI, potentially due to its deleterious effects on ferroptotic regulation.

### 8.6. Targeting Ferroptosis in Heart Failure

There is evidence supporting the use of iron chelators for the treatment of HF. For example, deferoxamine decreased infarct size, ACSL4, and Fe^2+^ in a rat model of I/R injury, while GPX4 was significantly elevated [332]. Similarly, in mice subjected to I/R injury, deferasirox treatment also reduced lipid peroxidation, indicated by decreased 4-hydroxynonenal expression and the area at risk following infarction [333]. Ferrostatin-1, an inhibitor of lipid peroxidation, showed beneficial effects for the treatment of HF. Mice with HFpEF treated with ferrostatin-1 exhibited an increase in GPX4 and xCT expression, correlating with a decrease in iron levels and ameliorating cardiac dysfunction [334]. A clinical trial has determined that ferrostatin-1 improves outcomes following percutaneous coronary intervention in patients with coronary heart disease, although further research is required to determine if this drug is effective in ameliorating HF progression in humans [335]. Despite garnering less attention, liproxstatin is a drug that also inhibits lipid peroxidation and decreases ferroptotic cell death in mice subjected to I/R injury, as indicated by increased GPX4 expression and decreased ROS and infarct size [336].

### 8.7. Pyroptosis in Cardiac Physiology

Pyroptosis is a form of inflammation-driven programmed cell death in which danger signals trigger the formation of cell membrane pores by oligomerization of gasdermin (GSDM) fragments, thus allowing for the passive secretion of pro-inflammatory cytokines, including IL-1β and IL-18. Additionally, these pores allow for the abnormal osmosis of water into the cell and the transmembrane movement of ions, causing a disruption in the fragile ionic gradient and ultimately leading to cell swelling and bursting [337]. 

### 8.8. Pyroptosis in Heart Failure

Pyroptosis is clearly implicated in the pathogenesis of HF as many studies on human hearts have revealed an increase in caspase-1, IL-1β, and IL-18, correlating with the development of the disease [338,339]. Interestingly, human HFpEF patients exhibited significantly higher levels of serum IL-1β and IL-18 in comparison to those with HFrEF, suggesting a greater degree of pyroptosis in the former [340]. Likewise, in a rat model of DCM, cardiac expression of caspase-1, IL-1β, and IL-18 was also raised [341]. In murine models of acute infarction and pressure overload, cardiomyocytes exhibited a similar increase in IL-1β in addition to elevated apoptosis-associated speck-like protein containing CARD (ASC) and GSDM-D expression and caspase-1 activation, which is also indicative of more pyroptosis [342,343]. 

The role of nucleotide-binding oligomerization domain-like receptor protein 3 (NLRP3) inflammasome has been implicated in the pathogenesis of HF. Human HFpEF patients exhibited significantly elevated NLRP3 expression [340]. Similarly, in mice exposed to TAC, expression of NLRP3 was increased in comparison to controls [343]. Furthermore, mixed lineage kinase 3 (MLK3) expression was increased, while inhibition of MLK3 ameliorated cardiac dysfunction and decreased NLRP3, IL-18, and IL-1β, indicating a correlation between MLK3 and pyroptosis [344]. 

Absent in melanoma 2 (AIM2) is another protein that forms an inflammasome complex and is found to play a role in HF via mediating pyroptosis. The AIM2 inflammasome consists of AIM2, ASC, and pro-caspase-1 and is activated by cytosolic dsDNA released by bacterial pathogens or damaged mitochondria. Studies in human HF patients and animals with chronic HF found a positive correlation between disease progression and both AIM2 and IL-1β expression [345]. Studies in mice with DCM revealed significant upregulation of AIM2 correlating with increased cardiac M1 macrophage infiltration [346,347]. This effect was ameliorated in AIM2 knockout mice, and the progression of HF was delayed and associated with reduced IL-1β levels [346]. Similar results have also been observed in rats with DCM, wherein AIM2 expression was elevated when HF was present, while silencing of AIM2 inhibited HF development [348]. 

### 8.9. Targeting Pyroptosis in Heart Failure

Colchicine is an anti-inflammatory drug that inhibits the assembly of the NLRP3 inflammasome. In humans, post-MI treated with colchicine exhibited a significant reduction in subsequent ischemic events [349]. Furthermore, the Colchicina en Insufiencia Cardiaca Aguda trial determined that colchicine treatment of individuals with acute HF significantly reduced other pro-inflammatory cytokines IL-6 and CRP [350]. Additionally, mice with dilated cardiomyopathy displayed an increase in EF and a reduction in fibrosis following colchicine treatment [351]. A phase 1B clinical trial has indicated that treatment with the NLRP3 inhibitor dapansutrile significantly improved EF in humans with HFrEF [352]. MCC950 is a similar inhibitor that acts by reconfiguring NLRP3 into a closed conformation, thus antagonizing its pro-pyroptotic activities [353]. In mice, MCC950 treatment was able to ameliorate the cardiac dysfunction triggered by both isoproterenol-induced HF and HFpEF. These findings correlated with a decrease in NLRP3, IL-18, IL-1β, caspase-1, and GSDM-D expression [340,354]. 

Inhibition of caspase-1 with the specific antagonist VX-765 has also been proven to be beneficial in vivo. In both rat I/R and murine acute MI models, treatment with VX-765 significantly reduced infarct size, indicative of decreased cell death [355,356]. Similar results have been observed in rats subjected to left anterior descending artery ligation in which VX-765 also ameliorated MI-induced systolic dysfunction, fibrosis, and IL-1β secretion [356]. These promising data suggest that HF progression could be alleviated in humans by the inhibition of caspase-1 with VX-765, although clinical data in support of this have not yet been acquired.

## 9. Endothelial Dysfunction

### 9.1. Endothelial Function in Cardiac Physiology

The heart also comprises endothelial cells that account for the third most abundant cell type in the mammalian heart [357]. Since the distance between the endothelial cell and the neighboring cardiomyocyte is approximately 1μm, there is an intimate relation and intercellular dependence between endothelial cells and cardiomyocytes under physiological as well as pathological states [358]. Endothelial dysfunction in recent years has served as an important prognostic marker for HF initiation as well as progression in both HFpEF and HFrEF patients [359]. Clinical studies have also shown a positive correlation between the degree of endothelial dysfunction and the severity of HF, thereby predicting mortality risk [359,360]. 

**Figure 4:**
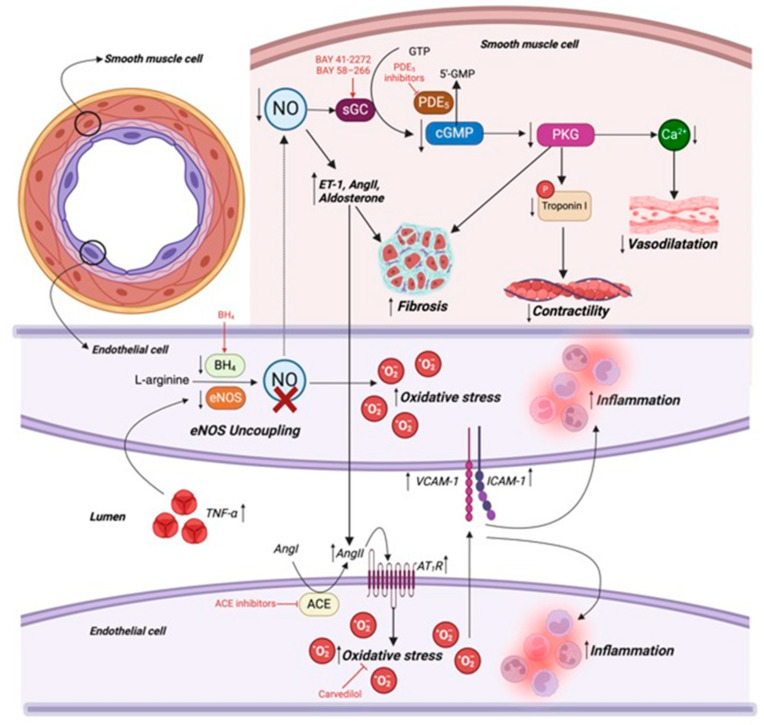
An overview of the mechanisms leading to endothelial dysfunction in heart failure. In a healthy heart, nitric oxide (NO) released by the catalytic activity of endothelium nitric oxide synthase (eNOS) regulates vascular function. However, the different molecular pathways contributing to reduced NO bioavailability lead to endothelial dysfunction-induced heart failure. Potential treatments are indicated in red. ACE, Angiotensin-converting enzyme; ANGI, Angiotensin I; ANGII, Angiotensin II; AT_1_R, AT_1_ angiotensin receptor; BH_4_, Tetrahydrobiopterin; cGMP, Cyclic guanosine monophosphate; eNOS, Endothelium nitric oxide synthase; ET-1, Endothelin-1; GMP, Guanosine monophosphate; GTP, Guanosine5′-triphosphate; ICAM-1, Intercellular cell adhesion molecules-1; NO, Nitric oxide; PDE_5_, Type 5 phosphodiesterase; PKG, Protein kinase-G; sGC, Soluble guanylate cyclase; TNF-α, Tumor necrosis factor-α; VCAM-1, Vascular cell adhesion molecules-1.

Endothelium covers the inner wall of blood vessels. It serves as a structural and functional barrier between the blood and the vessels, thereby preventing leucocyte and platelet adhesion, regulating permeability to plasma, and maintaining blood flow [361]. Under normal physiological conditions, healthy endothelial cells release vasodilators and vasoconstrictors in response to different stimuli in a balanced manner, which helps exert a vasoregulatory effect [362]. Nitric oxide (NO), released via endothelium NO synthase (eNOS) enzymatic activity, is one of the predominant regulators of vascular function. eNOS needs redox-sensitive cofactor tetrahydrobiopterin (BH_4_) as an electron donor to synthesize NO. NO diffuses into the smooth muscle cells of the vessel wall, causing vasodilatation in response to endogenous factors like acetylcholine, bradykinin, ischemia, and mechanical stimuli, including shear stress (Figure 4) [363]. Additionally, the endothelium is also responsible for providing anti-proliferative and anti-inflammatory effects by mediating cytokine and adhesion molecule expression, regulating fibrinolysis and coagulation pathways that maintain normal hemostatic properties of the blood vessels by balanced production of anti/procoagulant factors [364].

### 9.2. Endothelial Dysfunction in Heart Failure

The failing heart is in an altered redox state that overproduces ROS, as mentioned above. The recent literature suggests that the abnormal cardiovascular phenotype seen in HF is mainly due to imbalances between cardiac oxidative stress and NO bioavailability [365]. In HF, increased neurohumoral activation, increased release of inflammatory cytokines, and shear stress promote atherogenesis and oxidative stress while simultaneously diminishing NO production [366]. Additionally, in HF, BH_4_ levels are reduced, driving eNOS to produce superoxide anion instead of NO, which is clinically known as eNOS uncoupling (Figure 4) [367]. This leads to a vicious cycle of increased oxidative stress and reduced NO bioavailability, which ultimately leads to the development and progression of HF [368,369]. Although eNOS uncoupling and reduced NO bioavailability-induced endothelial dysfunction are seen in both HF phenotypes, myocardial biopsies revealed its increased occurrence in HFpEF patients [370].

Recently, microvascular complications, including endothelial cell dysfunction, have been an emerging and much less appreciated contributor to HF. A clinical study showed that in HF, the endothelium-dependent vasodilatory response is blunted as measured by forearm blood flow fluctuations in response to methacholine both in HFpEF as well as HFrEF patients [371]. This effect is not only observed in coronary artery atherosclerosis but is also noted in diabetes, chronic kidney disease, and hypertension-mediated HF [358]. The endothelial function is differentially affected depending on the comorbidities. For example, in HFpEF, microvascular complications are predominant and severe compared to HFrEF [372,373]. Additionally, endothelial dysfunction occurs earlier in HFpEF, serving as an early marker rather than a later symptom in HFrEF [374]. In chronic HFrEF patients, impaired endothelial vasodilatation is an independent predictor of mortality and hospitalization [375]. However, in HFpEF patients, it is not evaluated adequately despite its severity [374]. 

Additionally, there are also gender-specific differences in the endothelial dysfunction associated with HF. In men, macrovascular dysfunction-induced HFrEF is common, while women are predisposed to microvascular dysfunction, including endothelial inflammation-induced HFpEF [376]. Moreover, women exhibit accentuated immune responses with increased levels of pro-inflammatory cytokines and enhanced activation of inflammatory T cells, which can also affect endothelial function, as discussed further [377]. 

Furthermore, several animal HF models have demonstrated sex-based differential NO signaling [378]. For instance, TAC induced rapid and stable neuronal NOS (nNOS) expression in male rats, while it was delayed in female rats [379]. Moreover, estrogen also plays a role in NOS expression post-TAC in ovariectomized and non-ovariectomized rats, with the former involved in nNOS and the latter regulating eNOS activity [380]. While estrogen has been hypothesized as being protective against different CVDs, the exact mechanisms of its endothelial protection, especially in HFpEF, are not well understood [381]. Therefore, it is crucial to understand the differences in mechanisms leading to endothelial dysfunction in both HFpEF and HFrEF in both genders for developing better personalized therapeutic strategies.

### 9.3. Mechanisms Underlying Endothelial Dysfunction in Heart Failure

Multiple mechanisms contribute to lower NO bioavailability in HF. For instance, multiple episodes of I/R lead to dysregulated NO pathways that induce metabolic alterations in the myocardium, causing HF [382]. Impaired coronary vasodilation post-ischemia occurs due to impaired eNOS activity [358]. Mechanistically, induction of ischemia diminishes the cardiac BH_4_ content in isolated rat hearts with time via a nicotinamide adenine dinucleotide phosphate (NADPH) oxidase-dependent mechanism [383]. This ultimately triggers eNOS uncoupling, as mentioned above, in the post-ischemic heart, leading to vascular oxidative stress [384]. Conversely, transgenic mice overexpressing eNOS attenuated the infarction post I/R [385], thereby solidifying the role of eNOS in improving endothelial dysfunction in CVDs. Additionally, diminished eNOS activity can also be triggered due to increased circulating cytokines, particularly TNFα, leading to progressive vascular deterioration, as seen in the serum of patients with severe HF [386,387]. Dysregulated NO pathways also affect myocardial contractility through impaired myocardial perfusion [358]. NO enhances LV relaxation by phosphorylating troponin I through cyclic guanosine monophosphate (cGMP)-dependent protein kinase pathway that increases diastolic LV distensibility [358]. However, transgenic mice with cardiac-specific NOS overexpression showed that with the increased presence of superoxide in the endothelium, myocardial susceptibility to cGMP-mediated desensitization is reduced, ultimately leading to myocardial contractile defects, as seen in HF [388]. 

Reduced NO levels in the endothelial cells also influence cardiomyocytes and fibroblasts. For example, reduced vascular NO downregulates the cGMP-PKG pathway in the cardiomyocytes as well, which affects collagen turnover in fibroblasts, causing increased LV remodeling, thereby increasing LV stiffness that eventually contributes to impaired relaxation in HF, especially in HFpEF [374]. Additionally, reduction in PKG levels leads to hypo-phosphorylation of titin, which is a cytoskeletal protein that also increases the stiffness of the cardiomyocytes, thereby contributing to the cardiomyocyte–endothelial crosstalk in HFpEF [374]. Additionally, diminished endothelial NO availability also promotes the proliferation of fibroblasts and myofibroblasts, along with increased collagen deposition [389] due to the increased profibrotic action of growth-promoting hormones like endothelin-1, AngII, and aldosterone [390]. Increased levels of endothelin-1 also accentuate vascular resistance, matrix production, and smooth muscle cell growth that results in vascular remodeling, endothelial dysfunction, and, ultimately, HF progression [390]. 

A recent emerging area of research that highlights the endothelial–fibroblast crosstalk in HF is endothelial-to-mesenchymal transition (EndMT), whereby endothelial cells lose their phenotype and gain mesenchymal properties [391]. Multiple mechanisms that include but are not limited to TGF-β signaling have been associated with aberrant EndMT in different CVDs like coronary artery disease (CAD) or peripheral artery disease-induced atherosclerosis [392], MI [393,394], and valvular heart diseases like mitral valve prolapse [395]. However, the exact mechanisms responsible for EndMT in HF are poorly understood. 

Another pathogenic mechanism involved in the progressive vascular decline in HF, especially HFpEF, is the reduction in microvascular rarefaction, whereby small blood vessels like arterioles and capillaries are lost [396,397]. This leads to a declined myocardial oxygen supply, causing cardiac perfusion failure [398]. Recently, microvascular endothelial inflammation has also gained clinical importance in mediating endothelial dysfunction [396,399] by reducing coronary flow reserve and myocardial perfusion [400,401,402] in HFpEF patients via increased expression of endothelial adhesion molecules like ICAM-1 and VCAM-1 (Figure 4) [403]. 

### 9.4. Hypertension in Heart Failure

The renin–angiotensin–aldosterone system (RAAS) is an important regulatory system that helps maintain blood pressure, volume, as well as vasculature. However, in hypertension, RAAS activity is significantly increased, which leads to excess vasoconstriction, fibrosis, and hypertrophy in the endothelial cells, leading to HF [404]. RAAS also initiates endothelial-specific cytokine-like effects that include migration, growth, and vascular remodeling via extracellular matrix reconfiguration [405]. AngII, angiotensin-converting enzyme (ACE), and aldosterone are key contributors to mediating RAAS. ACE, in particular, regulates the levels of AngII and the vasoactive peptide bradykinin [404]. Hypertension-induced disrupted RAAS causes an increase in ACE activity, which leads to profound AngII synthesis as well as augmented bradykinin degradation, leading to pressure and/or volume overload-induced cardiac injury and eventual HF [406,407].

AngII in endothelial cells is responsible for NO production via eNOS [408] through a specific receptor, AT_1_, which is abundantly present in the vascular cells. Hypertensive stress upregulates AT_1_ receptors and NO scavenging, thereby impairing endothelial relaxation and enhancing superoxide production via NADPH oxidase activation, eventually resulting in vascular dysfunction and HF [409,410,411]. 

### 9.5. Diagnosing Endothelial Dysfunction

Multiple biomarkers have been recognized to predict endothelial dysfunction in HF. For instance, a clinical study showed that elevated plasma levels of von Willebrand factor positively correlated with increased BNP levels in HF [412]. Additionally, increased levels of cellular adhesion molecules like E-selectin, ICAM-1, and VCAM-1 in the plasma have been recognized to identify diabetes and CAD-induced endothelial dysfunction [413,414]. Of note, recent clinical studies have identified an endogenous competitive inhibitor of NOS, Asymmetric dimethylarginine (ADMA), as a novel biomarker for cardiovascular diseases, including HF [415]. ADMA plasma levels were shown to be significantly elevated in the case-control studies in patients with different cardiovascular risk factors like obesity [416], hypertension [417], diabetes mellitus [418], and vascular inflammation [419] compared to healthy controls [420]. Another endothelial-specific marker that has gained importance in recent years is endothelial progenitor cells (EPCs) [421]. Animal studies have highlighted the role of EPCs in promoting neo-angiogenesis and maintaining vascular homeostasis in rat models of infarction-induced HF [422]. Lower EPC concentrations were noted in patients with higher cardiovascular risk [422], thereby serving as a novel biomarker of endothelial dysfunction in CVDs [421]. 

Additionally, mature endothelial cells and EPCs secrete extracellular vesicles (EndoEVs), which are responsible for maintaining the structural and functional integrity of the vascular function by mediating receptor activation [423,424], intravesicular cargo transport of lipids [425] and transfer of miRNAs [426]. A clinical study noted a positive correlation between EndoEVs levels and CVD risk factors like metabolic syndrome, hypertension [427], and acute coronary syndrome [428], thereby highlighting EndoEVs’ ability as a biomarker for endothelial dysfunction-induced CVD progression. 

Apart from the biomarkers, there are multiple imaging modalities to assess endothelial function both clinically and in vivo. Intracoronary infusion of acetylcholine was the oldest and most commonly used technique clinically, which is now replaced with flow-mediated vasodilatation (FMD) due to its non-invasive nature, efficiency, and ease of use [429]. In the setting of CVDs, FMD has provided insights into the role of endothelial dysfunction in HF. It was noted that FMD was significantly impaired in patients with cardiomyopathies, CAD, and HF [430]. Moreover, it also serves as an important prognostic tool for CVD diagnosis by using it as a means to classify CVD patients into low-, intermediate-, and high-risk categories [431].

Recently, new emerging non-invasive techniques like intravital microscopy have gained clinical significance, especially at the bedside [432]. Intravital microscopy allows for direct visualization of microvasculature, including endothelial function, leucocyte–endothelial interaction, the flow velocity of red blood cells, and indirect observation of glycocalyx thickness [433]. The first and second generations of intravital microscopes, orthogonal polarization spectral (OPS), and sidestream dark field (SDF) are now upgraded to incident dark field (IDF) imaging with improved visualization and resolution of smaller capillaries [434]. Intravital microscopy was shown to be correlated with microvascular perfusion along with the thickness of endothelial glycocalyx [435].

Another non-invasive, more accurate, and well-studied nuclear imaging method to assess myocardial blood flow (MBF), coronary endothelial function, and endothelial-dependent stress responses is Positron Emission Tomography (PET) scans, which uses intravenous injections of tracers (^15^Oxygen-labeled water, ^13^Nitrogen-ammonia, and ^82^Rubidium) for visualization [436]. PET can also help in identifying patients with a higher risk of hospitalization or CVD death in CAD patients [437], patients with cardiometabolic diseases [438], and HF, especially HFpEF patients [439].

### 9.6. Targeting Endothelial Dysfunction in Heart Failure

Different approaches have been used clinically to maintain endothelial function. Apart from the conventional drug therapies, which will be discussed further, lifestyle and dietary modifications have shown positive effects, especially in senescent vascular endothelial dysfunction [440]. In particular, exercise and a calorie-deficit diet reportedly increased NO bioavailability and eNOS activity along with a reduction in endothelial oxidative stress [441]. In addition, improved vasodilation and NO bioavailability in HF patients after 4 weeks of aerobic exercise regime suggested a positive correlation between exercise and endothelial function [442].

Owing to the important role of BH_4_ in eNOS activity and, therefore, endothelial health, multiple interventions have targeted BH_4_ supplementation as a measure to improve endothelial function in HF [443]. BH_4_ treatment showed positive endothelial effects in animal models of hypertension and diabetes by preventing eNOS uncoupling [444,445,446].

In human studies, BH_4_ administration augmented endothelium-dependent vasodilation in individuals with hypertension [447], CAD [448], as well as HF [449]. 

Other pharmacological drugs that improve HF survival by targeting endothelial function include ACE inhibitors, β-blockers, type 5 phosphodiesterase (PDE_5_) inhibitors, and soluble guanylate cyclase (sGC) activators. ACE inhibitors help in improving bradykinin levels and reducing oxidative stress [450,451]. Moreover, clinical studies have shown that vasodilating β-blockers, like carvedilol, had a positive effect on endothelial function [452] in HF by exhibiting an antioxidative activity [453]. PDE5 inhibitors improve vasodilation and NO bioavailability in HF [454]. Lastly, the sGC activators enhanced endothelial function by targeting the NO-cGMP signaling pathway and increasing the NO bioavailability by sensitizing sGC to NO [455]. Multiple animal studies with sGC-targeted drugs like BAY 41-2272 and BAY 58–266 have also shown promising results in HF models (Figure 4) [456,457]. These studies highlight the possibility of targeting endothelial function to improve HF outcomes, thereby providing new opportunities for novel drug development. 

## 10. Dysfunctional Cardiac Conductivity

### 10.1. Adrenergic Signaling and Calcium Handling in the Heart

**Figure 5:**
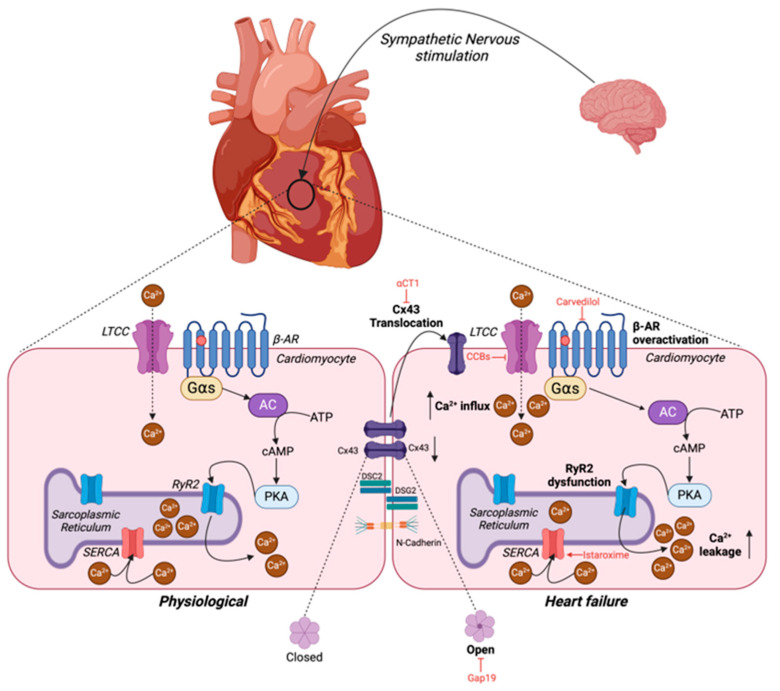
A representation of the abnormalities in mechanisms of cardiac contractility resulting in heart failure. Preservation of molecular pathways involving calcium (Ca^2+^) and adrenergic signaling, as well as cell junction proteins, are essential for maintaining cardiac contractility. In HF, these pathways are defective, as shown. Potential treatments are indicated in red. β-AR, β-adrenergic receptor; AC, adenylyl cyclase; ATP, Adenosine triphosphate; cAMP, second messenger cyclic AMP; CCBs, Ca^2+^ channel blockers; CX43, connexin43; DSC, Desmocollin; DSG2, Desmoglein-2; GαS, G_s_ alpha subunit; LTCC, L-type Ca^2+^ channels; PKA, protein kinase A; RyR2, ryanodine receptors type-2; SERCA, sarcoplasmic/endoplasmic reticulum Ca^2+^-ATPase.

The sympathetic nervous system (SNS), along with the parasympathetic nervous system, is one of two divisions of the autonomic nervous system [458]. These systems primarily work unconsciously in opposing manners to regulate various physiological functions throughout the body, including within the cardiovascular system. The former promotes cardiovascular activity, including increasing heart rate, strengthening myocardial contractility and peripheral vasoconstriction, while the latter depresses the cardiovascular system by decreasing heart rate [459]. These two nervous systems complement each other, regulating heart function both in physiology and pathology [460]. Cardiomyocytes expressing the β-adrenergic receptor (β-AR) are regulated by catecholamines released by the SNS, including epinephrine and norepinephrine (NE). β1-AR comprises 75%–80% of β-AR found in the heart, and once activated by synaptic NE or adrenergic agonist binding, it can couple to the Gαs protein and subsequently stimulate adenylyl cyclase (AC). This induces the production of second messenger cyclic AMP and, thus, the release of the catalytic subunit of protein kinase A (PKA), allowing it to phosphorylate Ca^2+^ channels (Figure 5). 

Following myocardial injury, the SNS is activated to enhance the β1-AR signaling pathway, thus increasing cardiac contractibility and output. However, when pro-excitatory effects on the heart persist over an extended period, they can lead to adverse outcomes for the cardiovascular system. In the past two decades, it has been discovered that β1-AR is involved in the regulation of many pathological functions. In HF patients, the mRNA and protein levels of β1-AR are consistently decreased. On the other hand, excessive β-adrenergic stimulation induces apoptosis of cardiomyocytes, which potentially contributes to the progression of myocardial failure and arrhythmogenesis [461]. Transgenic mice with cardiac-specific Gαs overexpression showed enhanced β-AR signaling through the Gαs/AC axis, leading to an initial improvement in cardiac function but ultimately causing long-term damage to the heart [462]. The importance of maintaining a stable equilibrium between over- and under-stimulation of β-ARs was further confirmed in cardiac-specific β1-AR overexpression mice that initially exhibited improved cardiac performance proceeded by the accelerated progression of HF [463], indicating that β1-AR was an important target for HF treatment [464]. 

The heart’s pumping ability is rooted in the regular and sustained contraction of cardiac muscle. The term cardiac contractility refers to the heart’s intrinsic ability to generate force and contract at a given speed [465]. Quantifying cardiac contractility primarily involves assessing the contractile performance of the sarcomere, the fundamental structural and functional unit of cardiomyocyte contraction. Sarcomere contraction is driven by Ca^2+^ dynamics, which regulate interactions between thin and thick filaments, primarily mediated by regulatory proteins such as troponin and myosin. The contraction of the muscles derives from the electrical stimulation, which is caused by altered intracellular Ca^2+^ levels (Figure 5). Increased contractility is primarily achieved by increasing Ca^2+^ influx or maintaining elevated Ca^2+^ levels in the cardiomyocyte cytoplasm during the action potential. Once a small amount of Ca^2+^ enters the cardiomyocytes through voltage-gated L-type Ca^2+^ channels (LTCCs), Ca^2+^-induced Ca^2+^ release (CICR) from the SR is triggered, and the Ca^2+^ exits the SR via ryanodine receptors type-2 (RyR2) channels. The SR is a membranous system in cardiomyocytes responsible for the regulation of Ca^2+^ dynamics and, consequently, the control of excitation–contraction coupling. The SR is necessary for the rapid clearance of Ca^2+^ via Ca^2+^-transporter SERCA from the cytoplasm to initiate muscle relaxation and contains a series of unique proteins, including calsequestrin, junction, and triadin. In addition to SERCA, any residual cytosolic Ca^2+^ can be sequestered by the mitochondria or is extruded via the sodium (Na+)/Ca^2+^ exchanger [466]. 

Abnormal Ca^2+^ signaling is identified as a form of pathological remodeling in HF. Under stressed conditions, cardiomyocytes can exhibit spontaneous Ca^2+^ release from the SR, primarily during diastole [467]. This means that Ca^2+^ release is not triggered by CICR due to LTCC-mediated Ca^2+^ influx; instead, it can result from RyR2 dysfunction or SR Ca^2+^ overload, leading to exacerbation of cardiac dysfunction in HF. RyR2 clusters progressively disperse during β-AR overactivation due to channel phosphorylation by PKA, diminishing the decreased efficiency of CICR, which implicates an important meaning for HF and other diseases with a risk of arrhythmia [468]. Moreover, RyR2 dysfunction has also been implicated in arrhythmic cardiomyopathy due to the remodeling of Ca^2+^ handling [469,470]. Thus, it is found that RyR2 stabilization therapy is effective in preventing LV remodeling and fatal arrhythmia for HF induced by MI in mice [471]. Additionally, a significant decrease in the efficacy of the Na^+^/Ca^2+^ exchanger has been observed in murine models of HF. Altogether, this results in an abnormally large amount of Ca^2+^ being taken up by the mitochondria, leading to mitochondrial Ca^2+^ overload, which contributes to oxidative stress and cytochrome c-driven apoptosis [466].

### 10.2. Physiology and Pathological Role of Sarcomeres in the Heart 

The sarcomere is the contractile element of cardiac muscle and, as previously mentioned, relies heavily upon Ca^2+^ signaling to provide synchronous contraction and relaxation of the myocardium. In brief, sarcomeres consist of alternating thick myosin and thin actin filaments in a striated manner, and it is the interaction and subsequent movement of these filaments that drive muscle contraction [472]. Tropomyosin forms a helical structure around the actin, covering the myosin binding sites, thus inhibiting their association. Troponins C, T, and I are located on the actin filaments and are responsible for regulating tropomyosin organization. Ca^2+^ binding to troponin C causes tropomyosin’s conformational change to reveal the myosin binding points on the actin filament. Troponin T interacts directly with tropomyosin and maintains its position on the actin filament. Finally, troponin I inhibits the interaction between troponin T and tropomyosin [472]. Many studies have identified a correlation between sarcomere function and HF and, as discussed in a comprehensive review by Hamdani et al., a common feature appears to be the inappropriate phosphorylation and dephosphorylation of the key sarcomeric proteins, which consequently affects their function [473].

### 10.3. Physiological and Pathological Roles of Junction Proteins in the Heart

The intercalated discs (ID) are highly specialized structures that are usually located at the long end of the cardiomyocytes. Different junction proteins, including GJs, desmosomes, and adherens junctions, form different types of junctional complexes that provide mechanical strength and electrical coupling to maintain cardiomyocyte morphology and electrical conduction (Figure 5).

#### 10.3.1. Gap Junctions

GJs form low-resistance pores between cells and are composed of protein subunits called connexins (Cx), of which there are up to 21 different types in humans [474]. A GJ channel comprises twelve Cx proteins, with each cell contributing six. These six Cx subunits form a hemi-channel known as a connexon in the plasma membrane. The connexon then docks with another connexon in the intercellular space to create a complete GJ channel [475]. 

Cx are transmembrane proteins with four membrane-spanning helices. As part of a large protein family, they exhibit functional diversity despite their close relation. In the ventricular myocardium, the predominant Cx isoform is Cx43. Alterations in Cx43 expression and distribution were observed in myocardial diseases, including hypertrophic cardiomyopathy, HF, and ischemia [476]. A guinea pig model displayed decreased Cx43 per myocyte at the compensated hypertrophy stage of congestive HF under pressure overload, which is the first time to show that Cx43 is reduced in mechanical overload-induced failing hearts [477]. In addition to Cx43, at least four other Cxs are present in the heart, namely, Cx30.2, Cx40, and Cx45. These specific proteins of the GJ are expressed in different parts of the heart, forming connections responsible for different functions. Cx40 is expressed de novo in atrial myocytes, the AV node, the bundle of His, and the ventricular conduction system [478]. Cx40 knockout results in AV block and bundle branch block. Cx45 expression is primarily localized to the SA and AV nodes. Heterozygous Cx45 deficiency does not alter AV conduction; however, in the absence of Cx40, Cx45 deficiency further exacerbates AV conduction delays, suggesting some interaction between the two Cx isoforms. Mouse Cx30.2 and its human ortholog Cx31.9 have been reported to be expressed in the vascular smooth muscle cells, the brain, and the testis. However, unlike Cx45, Cx30.2 has been reported to slow AV conduction in mouse hearts [479].

#### 10.3.2. Desmosomes

Desmosomes are a type of intercellular junction that appears as electron-dense plaques at the ID area and are well known for maintaining structural connections. Desmosomes consist of an intercellular and an intracellular part. The intercellular part is formed by the desmosomal cadherins desmocollin (DSC) and desmoglein (DSG), which interact in a heterophilic manner in the extracellular space to connect adjacent cells. The intracellular part of the desmosome consists of the proteins plakoglobin, plakophilin, and desmoplakin [480]. The intercellular part of the cardiac desmosome is built up by DSG2 and DSC2, both of which are single-pass transmembrane proteins. Among the two, DSG2 has attracted greater interest from clinicians and researchers as it is the only DSG expressed in the heart. Therefore, in the heart, it is unlikely that other DSG isoforms compensate for the loss of DSG2 function. 

DSG2 contains putative N-glycosylated sites, which are important for its normal function [481]. It has been proven that mutations in the glycosylation sites of DSG2 have been associated with weakened myocardial desmosomes in arrhythmogenic right ventricular dysplasia/cardiomyopathy (ARVD/C) [482,483]. The global DSG2 deletion is embryonically lethal in mice, while the deletion of a fragment within the first and second extracellular domains of murine DSG2 causes a similar cardiomyopathy phenotype [481,484]. Studies employing a transgenic mutant DSG2 mouse model, in which wild-type DSG2 alleles were replaced by mutant alleles coding DSG2 lacking parts of adhesive domains, exhibited a series of abnormalities, including dissociation of IDs and inflammation, and eventual ARVC with ventricular dilation, fibrosis, and cardiac arrhythmia [485]. These mice started to display a cardiac phenotype by four weeks that involved loss of viable cardiomyocytes and heavy cell calcification. Another transgenic model in which cardiac DSG2 was knocked out showed a loss of adhesive function at IDs in adult animals, although heart development was normal [486]. 

#### 10.3.3. Adherens Junctions

Adherens junctions (AJs) play a role as a primary anchor for myofibrils and connect actin filaments from adjacent cells, which allows the cell to retain shape upon mechanical stress and enables transmission of contractile force from one cell to another [487]. Furthermore, AJs are responsible for the initiation of the contractions of the myocardium and for sustaining cell proximity as the heart expands and contracts. They establish a robust mechanical linkage among cardiomyocytes by connecting themselves to the actin cytoskeleton, ensuring consistent mechanical resilience throughout the heart. AJs are situated in the zones of the ID perpendicular to the long axis of the myocyte to be able to transmit mechanical forces from one cell to another [488]. AJs consist of the transmembrane protein cadherins and intracellular component catenins, including p120-catenin, β-catenin, and α-catenin. Neural-cadherin (N-cad) is the main isoform of the cadherins protein superfamily in cardiomyocytes and plays the core role in AJs function. 

Disruptions to AJs have been implicated in a range of cardiac diseases. Dos Santos et al. investigated the alterations in the expression of ID proteins in the transition of compensated cardiac hypertrophy to dilated heart development in Wistar rats. They found that reduced expression of N-cad in hypertrophic and dilated hearts was associated with decreased cardiac function [489]. Kostetskii et al. proved that cardiac-specific N-cad depletion led to modest dilated cardiomyopathy before animals died of cardiac arrhythmic death due to the missing ID structure [490]. In addition to the above results, a study investigated the border zone of MI in rat hearts and found that N-cad expression and localization decreased but did not disappear, suggesting the potential function of N-cad in MI [491]. β-catenin was also found to be lost in myocardial hypertrophy and HF patients due to the lost association between estrogen receptor α and β-catenin [492]. 

### 10.4. Targeting Defective Cardiac Contractility in Heart Failure

Drugs targeting β-AR have been widely used in the clinical treatment of HF, especially β-AR blockers, which can directly block the action of catecholamines, therefore reducing the effect of SNS overdrive, resulting in increased EF and survival [493]. Moreover, it has been proven that β-AR blockers also increase cardiac mitochondrial respiration and inhibit oxidative stress by activating PPARs and limiting ROS production in cardiomyocytes [494,495]. Among all the β-AR blockers, carvedilol is a third-generation neurohormonal antagonist with the best usage to treat chronic HF, hypertension, and LV dysfunction. 

Drugs targeting the Ca^2+^ signaling pathways date back to the 1960s. Ca^2+^ channel blockers (CCB) are widely used to treat various conditions relating to cardiac function, mainly for hypertension or atrial arrhythmia [496]. Two different types of CCBs, dihydropyridines and non-dihydropyridines, have the same mechanisms of action but different effects on cardiac function. Both can inhibit Ca^2+^ entering the cardiomyocytes, which leads to vasodilation and lower blood pressure. Apart from traditional CCBs, some new therapeutic drugs have been reported, among which istaroxime might be a promising future candidate. It can increase the activity of SERCA2a, resulting in more Ca^2+^ entry into the SR. Clinical trials have seen that a 24-hour infusion of istaroxime significantly improved cardiac function without major adverse cardiac effects [497,498]. However, in a meta-analysis determining the efficacy of istaroxime for the treatment of acute HF, while cardiovascular function was consistently significantly improved, there was a significant increase in the prevalence of any adverse effects regardless of severity [499]. Additionally, a major disadvantage of istaroxime is that it is metabolized by the liver extremely quickly, leading to a notably short half-life. This means istaroxime is only suitable for acute treatment and is not appropriate for long-term use [500]. Rycals are another family of drugs that aim to reduce cytosolic Ca^2+^ via inhibiting the leak of Ca^2+^ through RyR2, thus offering the potential for the treatment of HF. While their efficacy has not yet been determined in humans, administration of the Rycal JTV519 to dogs with HF reduced LV remodeling and ameliorated the HF phenotype in conjunction with reduced Ca^2+^ leakage [501].

The sarcomeric function also provides an intriguing therapeutic avenue with clinical trials, indicating that this is a potentially beneficial target for the treatment of HF. In the Global Approach to Lowering Adverse Cardiac Outcomes through Improving Contractility in Heart Failure trial, patients with HFrEF were administered the drug omecamtiv mecarbil, which binds to the myosin filaments, increasing the myosin–actin-binding capacity and, thus, the contractile force of each power stroke. Individuals who received this drug exhibited reduced NT-proBNP levels in addition to fewer incidences of cardiovascular events or death [502]. Alternatively, levosimendan is a Ca^2+^ sensitizer, which interacts directly with troponin C-enhancing contractile force without the need to raise Ca^2+^ levels. There have been multiple trials indicating that levosimendan can ameliorate HF progression and alleviate symptoms across the HF disease spectrum, including those with acute HF and advanced HFrEF [503,504,505].

Therapies targeting GJs in IDs are not as widely used but include Gap19 and αCT1 [506]. The selective Cx43 hemichannel inhibitor Gap19 protects ventricular cardiomyocytes, reducing infarct size by one-fifth in mice and one-quarter in rat hearts during I/R. TAT-Gap19, with improved cell permeability, showed a three-quarter reduction in brain infarct size in a stroke model, suggesting potential for enhanced cardioprotection [507,508]. The Cx43-based peptide αCT1, when administered before ischemia, enhances LV function and protects against I/R injury in murine hearts. The αCT11 variant, a modified version of αCT1 without the antennapedia sequence, shows even greater cardioprotective effects [509]. 

Since DSG2 is responsible for the formation and maintenance of the cell structures, there are two strategies targeting DSG2 for the treatment of cardiomyopathies. Given that mutations in DSG2 can result in disruption of adhesion and tissue structural integrity, correcting the DSG2 gene mutation by using adeno-associated virus (AAV) gene therapy is a potential way in arrhythmogenic cardiomyopathy patients. Mikio et al. have proved that AAV-mediated gene replacement therapy to restore DSG2 before disease progression can be an effective upstream therapy [510]. 

Targeting AJs represents a promising therapeutic approach, given their critical role in cardiac function. However, the development of drugs specifically targeting AJs remains in its early stages. Devemy and Blaschuk identified N-cad antagonists, which are specific peptides with biological activity capable of influencing endothelial cell adhesion and morphology, highlighting their potential as templates for therapeutic drug development [511].

## 11. Conclusions

The molecular pathology of HF is clearly complex, featuring a vast array of defective signaling pathways culminating in increased hypertrophy, fibrosis, and cell death, thus inhibiting the heart’s function. We have broadly characterized these into eight subcategories: mitochondrial dysfunction, lipotoxicity, ER stress, autophagy, inflammation, programmed cell death, endothelial dysfunction, and defective cardiac contractility. These molecular mechanisms act independently or synergistically, thereby enhancing disease progression. Current treatment strategies are not sufficient to tackle HF in clinics. Here, we have summarized the molecular mechanisms and how their targeting could potentially improve outcomes in individuals with HF, although the feasibility and efficiency of the treatment options need further bench-to-bedside investigation.

## Data Availability

Not applicable.

## Abbreviations

The following abbreviations are used in this manuscript:

AngII	Angiotensin II
ATP	Adenosine Triphosphate
Ca^2+^	Calcium Ion
DCM	Diabetic Cardiomyopathy
EF	Ejection Fraction
eNOS	Endothelial Nitric Oxide Synthase
ER	Endoplasmic Reticulum
FA	Fatty Acid
FAO	Fatty Acid Oxidation
GA	Golgi Apparatus
GJ	Gap Junction
HF	Heart Failure
HFpEF	Heart Failure with Preserved Ejection Fraction
HFrEF	Heart Failure with Reduced Ejection Fraction
I/R	Ischemia/Reperfusion
ID	Intercalated Discs
IL	Interleukin
LV	Left Ventricle
MI	Myocardial Infarction
NF-κB	Nuclear factor kappa-light-chain-enhancer of activated B cells
NO	Nitric Oxide
OXPHOS	Oxidative Phosphorylation
PPAR	Peroxisome Proliferator-activated Receptor
ROS	Reactive Oxygen Species
SERCA	Sarcoendoplasmic Reticulum Calcium ATPase
SR	Sarcoplasmic Reticulum
TAC	Transverse Aortic Constriction
TCA	Tricarboxylic Acid Cycle
TG	Triglyceride
TNFα	Tumor Necrosis Factor α
UPR	Unfolded Protein Response
β-AR	β-Adrenergic Receptor
